# Mechanism of Inhibition of Human Islet Amyloid Polypeptide-Induced Membrane Damage by a Small Organic Fluorogen

**DOI:** 10.1038/srep21614

**Published:** 2016-02-18

**Authors:** Xiaoxu Li, Mingwei Wan, Lianghui Gao, Weihai Fang

**Affiliations:** 1 Key Laboratory of Theoretical and Computational Photochemistry, Ministry of Education, College of Chemistry, Beijing Normal University, Beijing 100875, China

## Abstract

Human islet amyloid polypeptide (hIAPP) is believed to be responsible for the death of insulin-producing *β*-cells. However, the mechanism of membrane damage at the molecular level has not been fully elucidated. In this article, we employ coarse- grained dissipative particle dynamics simulations to study the interactions between a lipid bilayer membrane composed of 70% zwitterionic lipids and 30% anionic lipids and hIAPPs with α-helical structures. We demonstrated that the key factor controlling pore formation is the combination of peptide charge-induced electroporation and peptide hydrophobicity-induced lipid disordering and membrane thinning. According to these mechanisms, we suggest that a water-miscible tetraphenylethene BSPOTPE is a potent inhibitor to rescue hIAPP-induced cytotoxicity. Our simulations predict that BSPOTPE molecules can bind directly to the helical regions of hIAPP and form oligomers with separated hydrophobic cores and hydrophilic shells. The micelle-like hIAPP-BSPOTPE clusters tend to be retained in the water/membrane interface and aggregate therein rather than penetrate into the membrane. Electrostatic attraction between BSPOTPE and hIAPP also reduces the extent of hIAPP binding to the anionic lipid bilayer. These two modes work together and efficiently prevent membrane poration.

Plaques of human islet amyloid polypeptides (hIAPP) have been observed in pancreatic *β*-cells in more than 90% of type II diabetes patients^1^,^2^. This 37-amino acid peptide is co-secreted in response to the same stimuli that lead to insulin release^2^. This peptide is believed to be responsible for the damage to the insulin-producing *β*-cell and eventually type II diabetes. Several mechanisms of IAPP-induced cytotoxicity have been proposed; for example, in one mechanism, membrane pores are initially induced by intermediate-sized peptide aggregates^3^,^4^,^5^,^6^,^7^; in another mechanism membrane damage occurs during the growth of IAPP fibrils^8^,^9^,^10^. Therefore, quite abundant inhibitors, including organic molecules^11^,^12^,^13^, fragments of hIAPP^14^,^15^,^16^, and variant native proteins and their fragments^17^,^18^,^19^,^20^,^21^,^22^ have been tested by characterizing their activities and the mechanism of inhibition of fibril formation or cytotoxicity.

To search for therapeutic agents that can rescue hIAPP-induced cytotoxicity, it is necessary to uncover the mechanism of amyloidal self-assembly and monitor the kinetic conformation change induced by inhibitors. Experimental studies on the interactions between model membrane and IAPP provide important implications for native physiological functions of IAPP. For example, sequence and spectroscopic comparisons of nonamyloidogenic rodent IAPP (rIAPP) and amyloidogenic hIAPP proposed a general mechanism for the formation of membrane-bound *α*-helical aggregates^4^. The *α*-helical aggregated states induced membrane disruption in the early stage and correlate with the fiber formation by hIAPP in the later stage. The same group also observed that IAPP induced all-or-none liposome leakage^5^, which suggested that there were two oligomeric classes: the leakage-incompetent and the leakage-competent states. Conversion of leakage incompetence to competence was suggested as a consequence of conformation change. The conversion rate was dependent on the peptide concentration. Such IAPP-induced membrane leakage and cell death were identified through a common mechanism as antimicrobial peptides^23^. To determine which residues are essential for the cytotoxicity and amyloidogenesis of the peptide, fragments of IAPP have also been studied^24^,^25^. The hIAPP_1–19_ fragment was found not forming amyloid fibers when incubated with membrane^24^. However, hIAPP_1–19_ induced membrane disruption to a near identical extent as the full-length peptide at low peptide concentration; while at higher peptide concentration, this fragment induced a greater extent of membrane disruption than the full-length peptide. In contrast, hIAPP_20–29_ was sufficient to form amyloid fibers^25^. Both rIAPP_20–29_ and hIAPP_20–29_ caused considerable membrane disorder. However, the disorder was not linked to the substantial membrane disruption. Cao, *et al*., further used series of mutations of hIAPP and rIAPP to test the roles of specific residues in membrane interactions^7^. They found that aromatic residues were not required for membrane damage, but the membrane damage was sensitive to the protonation state of histidine in the helical region. Based on the hypothesis that the membrane-bound *α*-helical IAPP oligomers represented the toxic species and are on pathway to amyloid formation, various amyloid inhibitors have been screened to identify their ability of inhibition^12^,^13^,^14^,^15^,^16^,^17^,^18^,^19^,^20^,^21^,^22^, especially the ability to bind with the *α*-helical states of hIAPP^11^.

Although much attention has been paid to the equilibrium structural analyses of the amyloid structure itself, as well as the hIAPP-inhibitor complexes using various experimental methods, such as solid-state NMR, X-ray crystallography, and optical techniques^26^, few techniques are able to track the structural details of the intermediates with both high structural and temporal resolutions. The features that control pore formation and fibril growth are not yet fully understood. Molecular simulation is a powerful alternative tool for providing structure and dynamics details that cannot be easily probed experimentally. All-atomic (AA) and coarse-grained (CG) molecular dynamic (MD) simulations have been applied to study the hIAPP folding in solution or the conformations of IAPP in a membrane^27^,^28^,^29^,^30^,^31^. Most AAMD studies of peptide-membrane interaction focused on the conformational equilibrium of IAPP with the initially given monomeric or oligomeric states, where the population of the oligomers and the variation of the membrane structure cannot be determined^29^,^30^. The self-assembly of IAPP oligomers in a lipid bilayer was recently simulated using the CG method^31^. However, in that work, the peptides were initially inserted into the membrane. The mechanisms driving the peptide to bind and trigger damage to the membrane are still not clear. For the hIAPP-inhibitor complexes, only very limited molecular modeling has been reported^22^, where only one inhibitor molecule binding to a single hIAPP peptide was simulated. To address the mechamisms of hIAPP-induced membrane damage and the function of a potent inhibitor at the molecular level, in this work, we employ coarse-grained dissipative particle dynamics (DPD) simulations to study the interactions between hIAPP, a small organic fluorogen molecule named sodium 1,2-bis[4-(3-sulfonatopropxyl)phenyl]-1,2-diphenylethene (BSPOTPE)^32^,^33^,^34^, and a anionic lipid bilayer with peptides initially randomly distributed outside the membrane.

HIAPP is an unstructured polypeptide in a solution; however, upon binding to the membrane, this peptide folds to an *α*-helical conformation under certain conditions^3^,^4^,^5^,^6^,^7^. The membrane-bound helical hIAPPs can further associate and convert to *β*-sheet-rich amyloid fibrils^8^,^9^,^10^. Though fiber state represents the product of amyloidogenesis, the *α*-helix containing intermediate seems to represent a toxic species that has the capacity to render model membrane permeability^35^. In this work, we perform simulations of full-length hIAPP as well as its fragments with *α*-helical structures to study the assembly process and its subsequent effect on the membrane. We find that hIAPPs associate with dimeric, trimeric, and even oligomeric structures when they bind to the membrane via an electrostatic attraction. Sufficient hIAPP molecules binding to the membrane can induce multiple toroidal-like pores in the membrane and cause growing membrane leakage. We demonstrated that the key factor controlling pore formation is the combination of peptide charge-induced electroporation and peptide hydrophobicity-induced lipid disordoring and membrane thinning. According to these mechanisms, to protect the membrane from hIAPP-induced damage, a potent inhibitor should have the capability to neutralize the cationic charges and shield the hydrophobic portions of the peptides. We suggest that a water-miscible tetraphenylethene BSPOTPE is an appropriate agent. This small organic fluorogen molecule was first synthesized as a bioprobe to monitor amyloidosis kinetics^32^,^33^. It was later discovered that this fluorogen molecule can also inhibit the nucleation of insulin and impede protofibril formation^34^. In this work, we investigate its potential inhibition effects on hIAPP-induced cytotoxicity. Our simulations show that a single BSPOTPE molecule can adsorb multiple hIAPP molecules to form oligomers. The oligomers further aggregate and elongate to prefibril-like structures. The hIAPP-BSPOTPE clusters have micelle-like structures that have separated hydrophobic cores and hydrophilic shells. Such kernelled amphiphilic configurations promote retainment of the hIAPP-BSPOTPE clusters in the water/membrane interface rather than their penetration into the membrane. The electrostatic attraction between BSPOTPE and hIAPP also reduces the extent of hIAPP binding to the anionic lipid bilayer. Additionally, the anionically charged nature of BSPOTPE decreases the electric potential across the bilayer induced by hIAPP and eventually prevent electroporation.

## Results

### *α*-helical hIAPP oligormers induce permeable pores in lipid membranes

**Figure 1:**
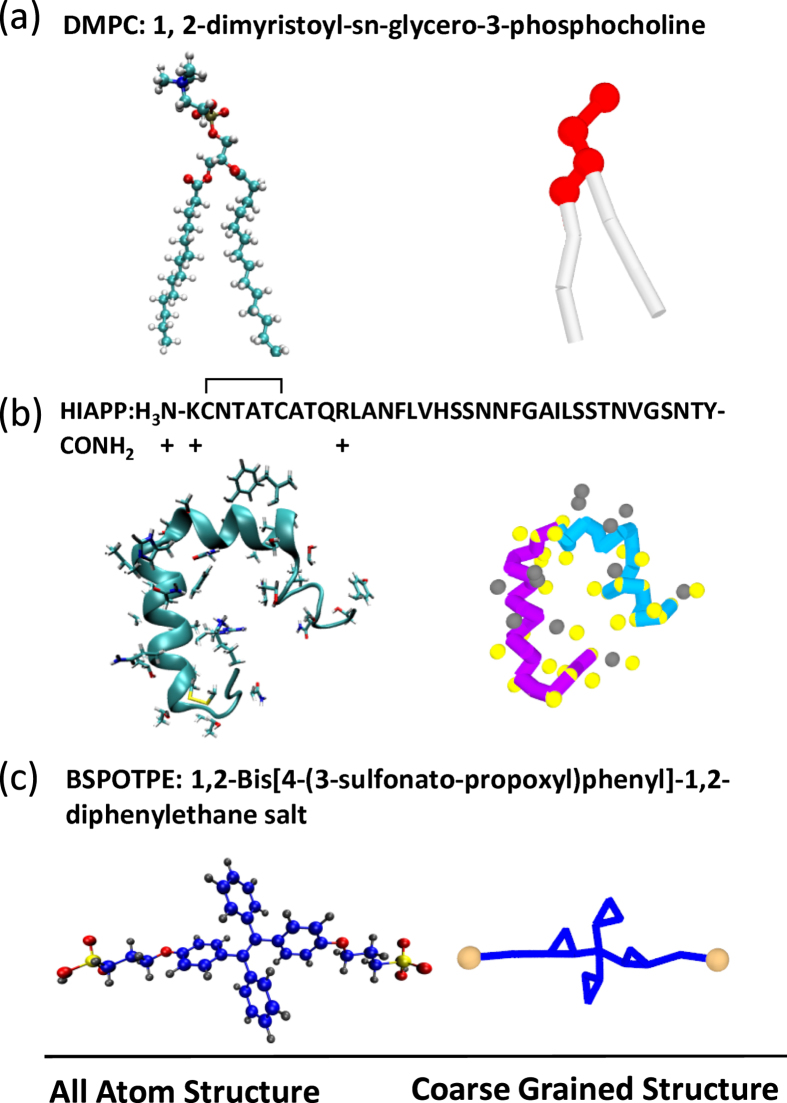
Atomic and coarse-grained structures of (**a**) DMPC, (**b**) hIAPP, and (**c**) BSPOTPE molecules. In the CG lipid model, the red beads represent the hydrophilic head groups and the gray bonds represent the hydrophobic tails. The CG back-bone of hIAPP is represented by magenta (residues 1–19) and blue (residues 20–37) bonds. The side-chain hydrophilic beads of hIAPP are yellow, and the hydrophobic beads are gray. The hydrophobic beads of BSPOTPE are blue, and the charged hydrophilic beads are gold.

We first simulated the assembly of 16 to 121 hIAPP molecules and a bilayer membrane composed of 1600 lipid molecules. The CG models of the molecules simulated here are given in Figs 1 and S1–S3 in the Supporting Information (SI). To simulate typical experimental conditions that can ensure peptide binding, the bilayer consists of 70% zwitterionic lipids [resembling dimyristoyl phosphatidylcholine (DMPC) lipids] and 30% anionic lipids [resembling dimyristoyl phosphatidylglycerol (DMPG) lipids]. HIAPP molecules are initially uniformly placed on square grids approximately 2 nm above the upper leaflet of a pre-relaxed bilayer membrane, as shown in Fig. S4.

**Figure 2:**
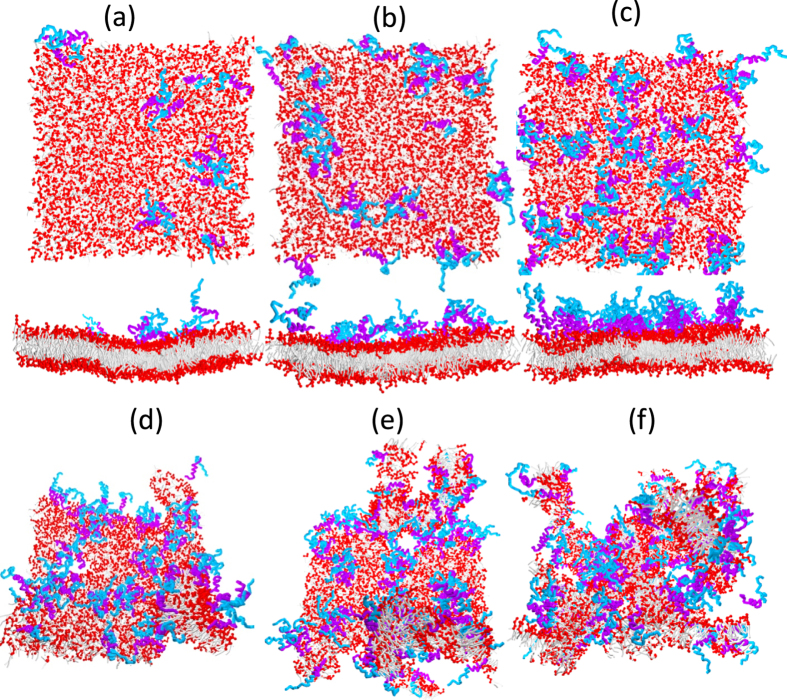
Snapshots of (**a**) 16, (**b**) 36, (**c**) 64, (**d**) 81, (**e**) 100, and (**f**) 121 *α*-helical hIAPP molecules interacting with a bilayer membrane composed of 1600 lipids at a simulation time of 1.144 *μ*s. Both top and intersectional views of the complexes are given at low peptide concentrations in (**a**), (**b**), and (**c**) to illustrate the binding states of hIAPP.

**Figure 3:**
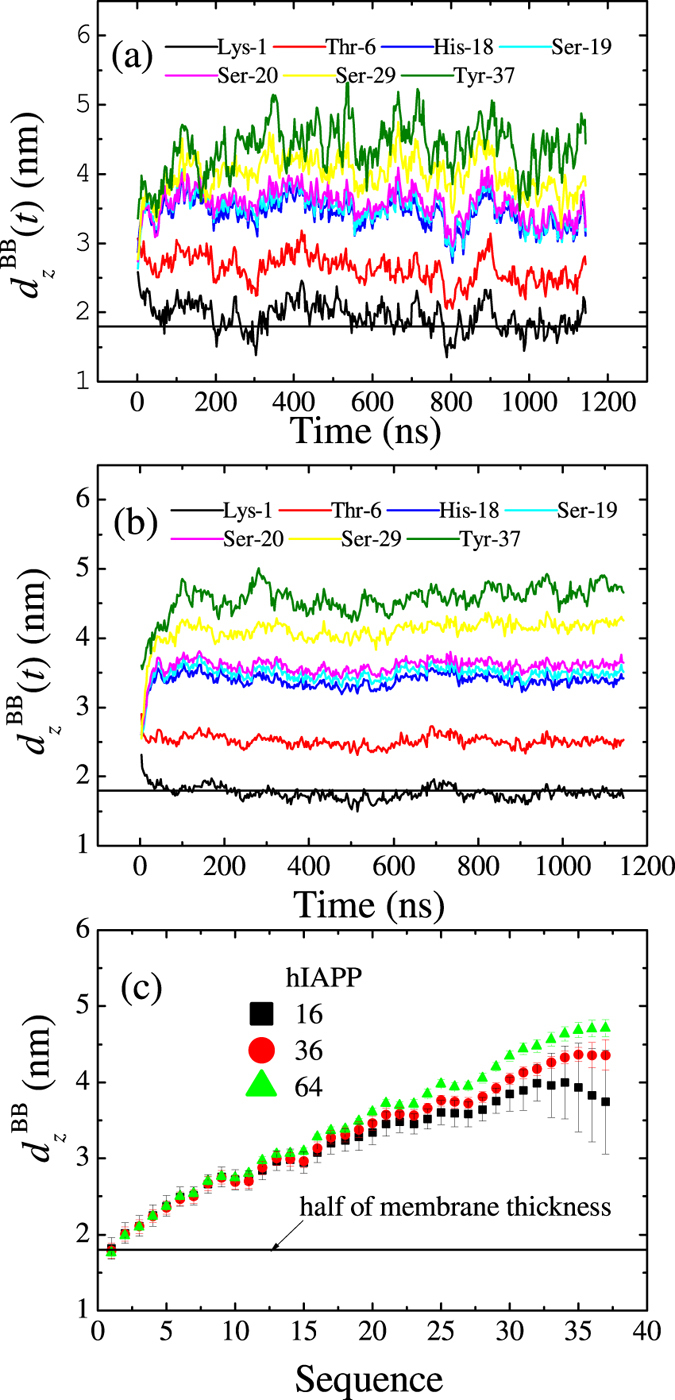
Time-dependent distances 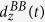 between typical hIAPP back-bone beads and the membrane center obtained from systems containing (**a**) 16 and (b) 64 hIAPP molecules. (**c**) Time-averaged distances 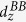 for all of the back-bone beads obtained from systems containing variou number of hIAPP molecules.

Figure 2 provides the snapshots of typical configurations of hIAPP-membrane complexes at various peptide/lipid (P/L) molar ratios. At a concentration P/L < 5/100, the peptides are fully adsorbed onto the surface of the membrane, mainly via electrostatic attractions between the cationic residues (Lys-1 and Arg-11) and anionic DMPG head groups. At a low peptide concentration [Fig. 2(a,b)], hIAPP monomers and dimers are commonly observed. Like antimicrobial peptides (AMPs)^36^,^37^, the amphipathic nature of hIAPP drives its hydrophobic face to penetrate into the membrane interior, while the hydrophilic face extends into the water solvent. In this way, the axes of the helical monomeric hIAPP are almost parallel to the membrane surface. However, unlike many AMPs, which are usually highly charged and have large hydrophobic portions (for example, Magainin 2 has 6 positive charges and 60% hydrophobic residues^36^), one hIAPP contains only 3 positive charges, mainly located near the N-terminal region, and 40% hydrophobic residues. These features make the parallel binding state of monomeric hIAPP not as stable as AMPs. As shown in Fig. 2(c), when the peptide concentration increases, hIAPP favors associating as dimers or trimers that have the N-termini attached to the lipid heads; however, the C-termini do not interact with the membrane. The binding affinity of hIAPP to the membrane and their orientations can be identified quantitatively by measuring the normal distance 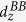 of the back-bone beads from the membrane center (see the Method section). Accordant with the snapshots in Fig. 2, both of the time-dependent 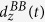 and time-averaged 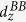 in Fig. 3 show that the distance between the N-termini and the membrane center is almost half of the membrane thickness, while the C-termini are the most distal residues to the membrane. Due to the aggregation, the overall distance between hIAPP and the membrane slightly increases with the peptide concentration. The two helices (residues 6–18 and residues 20–29) tilt with respect to the membrane. If we assume that the helices are rigid, the tilt angles are approximately 30 degrees for the helix in the N-terminal region and 25 degrees for the helix in the C-terminal region. The distance between hIAPP and the membrane also shows that at low peptide concentration where the monomeric state of hIAPP dominates, the C-termini of hIAPP are very flexible; while, at high peptide concentration, the aggregation suppresses the flexibility. The monomeric assemblies at low peptide concentration and oligomeric assemblies at relatively high peptide concentration are consistent with the experimental observations^4^,^5^.

**Figure 4:**
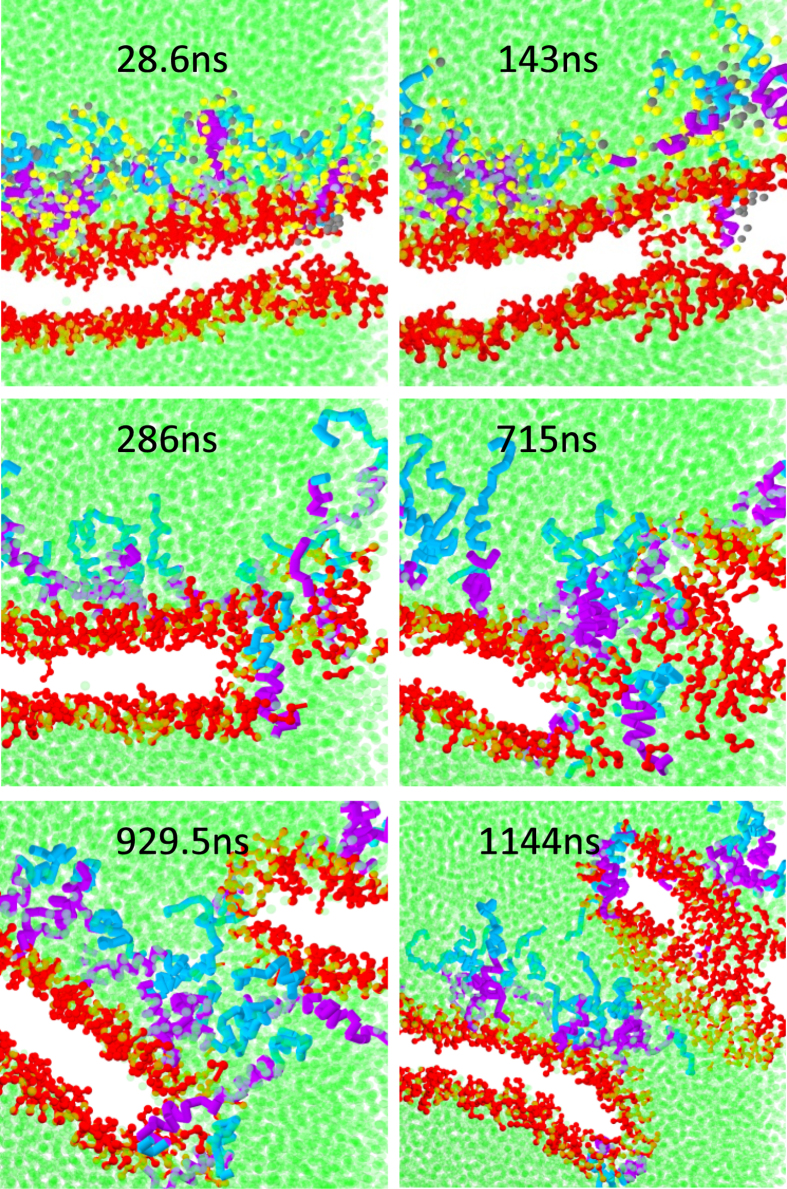
Evolution of a pore induced by 81 α-helical hIAPP molecules in a bilayer membrane. Lipid tails are invisible for clarity. Water beads in green are shown explicitly. The side-chain beads of hIAPP are presented early in the snapshots to illustrate the effects of amphipathicity, but these beads are invisible later to improve the clarity of the pore.

**Figure 5:**
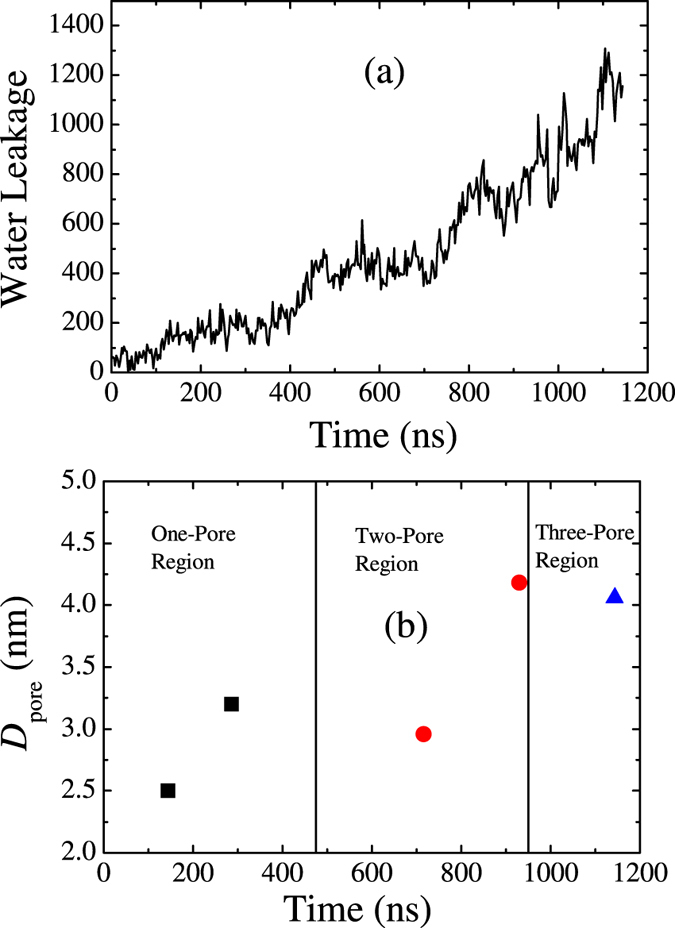
(**a**) Kinetic profile of membrane leakage induced by 81 *α*-helical hIAPP molecules. (**b**) Inner diameter of membrane pore 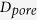 detected at various simulation time.

When the P/L molar ratio increases to 5/100 or more, helical hIAPP induces the formation of permeable pores, as shown in Fig. 2(d–f). Figure 4 shows the time evolution of a pore simulated at P/L ≈ 5/100. (The time evolution of the whole peptide-membrane complex corresponding to this figure is given in Fig. S4 in SI.) We find that the pore starts with one hIAPP and a few lipid head groups inserted into the hydrophobic core of the membrane. Later on, more peptides and lipid head groups are able to enter into the pore and enlarge the pore to an intermediate-sized stable pore composed of 3–6 peptides. Water, lipids, and even hIAPP itself can transport across the pore. At an even higher peptide concentration, more pores are able to form. Some of these pores can even fuse and lyse the membrane into micelle-like structures as shown in Fig. 2(e,f). The micelles are composed of both lipids and peptides. The disruption by helical hIAPP on the membrane is similar to the pores induced by the antimicrobial peptide Magainin 2 and resembles the carpet model^36^. Figure 5(a) presents the kinetic profiles of membrane leakage induced by 81 hIAPPs (see the Methods Section). The profile demonstrates that an *α*-helical hIAPP-induced water leakage increases monotonically and quickly, which resembles the first phase of the kinetic leakage profile measured in experiments^7^,^9^. This agreement implies that early in the process, the helical hIAPP oligomers are the effective species in porating the membrane. If we assume that a water pore is cylindrical, then the average inner diameter of a pore 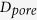 can be estimated by 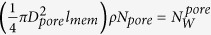, where 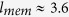 nm is the membrane thickness, 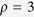 is the water density, 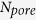 is the number of pores formed in the membrane patch, and 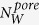 is the counted membrane leakage given in Fig. 5(a). Figure 5(b) presents the pore diameter obtained at various simulation time corresponding to the snapshots in Fig. 4. On average, the pore has inner diameter of 2–4 nm, which is comparable to the pore size detected by atomic force microscopy (AFM) image^6^ that has diameter of 1–2 nm. Because the pore has irregular rather than regular cylindrical shape, the estimation of the pore size here is relatively rough. Overall, the hIAPP-induced membrane pore formation is similar to that observed in experiments^4^,^5^: (i) The leakage is dependent on the peptide concentration; (ii) The leakage-competent state is an *α*-helical oligomeric aggregate; (iii) The leakage-competent state is capable of incorporating additional protein precursor and form stable pore.

**Figure 6:**
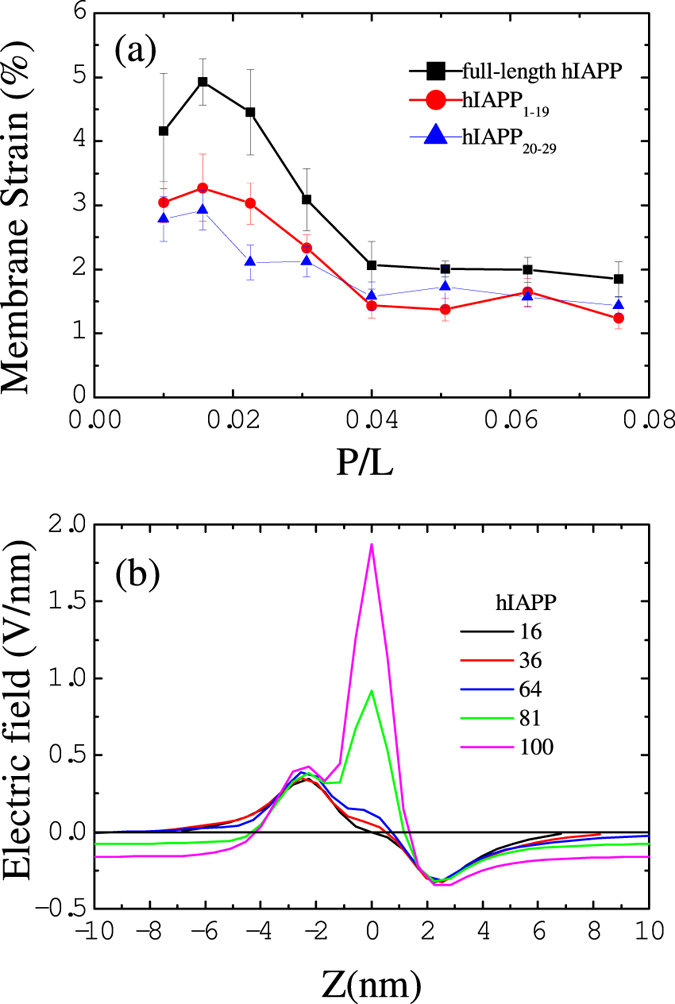
(**a**) Membrane strain induced by the binding of hIAPP and its fragments at various P/L ratios. (**b**) Normal component of the electric field as a function of distance Z from the membrane center caused by full-length hIAPP.

Like AMPs, helical hIAPPs have well separated amphiphilic portions; upon binding to the membrane, the hydrophobic portions penetrate into the hydrophobic core of the bilayer and induce compression on the peptide-rich leaflet of the membrane^23^,^36^,^37^. To relax the compression, the area of the membrane will increase, which eventually causes tension on the peptide-free leaflets. Such effects can be observed in Fig. 6(a), where we plot the membrane strain, i.e., the percentage change in the membrane area induced by hIAPP binding as a function of the P/L molar ratio (see the Methods Section). We noticed that the area of the membrane is more stretched at a low hIAPP concentration rather than high concentration. This is because helical hIAPPs have better separated amphiphilic portions and less ability to associate at low concentrations. Therefore, their hydrophobic portions favor penetrating into the membrane and compress the surrounding lipids. As the peptide concentration near the membrane increases, hIAPPs associate as dimers or trimers such that parts of hydrophobic residues are buried inside the multimers; thus, they have lower efficiency in perturbing the membrane. Such effect was already demonstrated by the distance between hIAPP and membrane in Fig. 3. We find that the membrane strain induced by hIAPP is less than 5% at any peptide concentration. Such change is less than the critical value at which membrane poration can be induced. (See the elastic properties discussed in the Supporting Information).

Despite the fact that hIAPP-induced membrane disorder and tension might not be sufficient to trigger the membrane permeabilization independently, we find that the cationic character of hIAPP can increase the electric potential across an anionic membrane and cause poration of the membrane^37^,^38^. To verify this effect, we calculate the normal component of the average electric field as a function of the distance Z from the middle of the membrane as given in Fig. 6(b). Data are obtained by averaging the electric field in the 20 ns before pore formation. The calculations show that when more than 81 hIAPP molecules (P/L > 5/100) bind to the surface of the membrane, the induced electric field exceeds the critical value of 0.4 V/nm at which pore formation by an external electric field can be induced^39^. These results suggest that peptide-induced electric and mechanical tension work together to propel lipid head groups and hIAPPs themselves to insert into the membrane and form permeable pores to release this tension. The critical peptide concentration that can trigger pore formation depends on the charge density of the membrane. For example, for pure DOPG membrane, hIAPP at concentration P/L ≈ 1/100 is sufficient to induce membrane leakage in experiments^4^. While our simulations are performed at anionic lipid concentration of 30% (within the typical physiological range), therefore, relatively higher critical peptide concentration (P/L ≈ 5/100) is observed.

We also perform simulations of hIAPP_1–19_ and hIAPP_20–29_ fragments interacting with bilayer membranes. The fragments are assumed to have the same *α*-helical conformations as they are in the full-length hIAPP. The snapshots in Fig. S5 show that hIAPP_1–19_ fragments aggregate in a less extent than full-length hIAPP, but disrupt the membrane in a similar manner as the full-length hIAPP. The binding of hIAPP_1–19_ fragments to the membrane induces relatively less strain than the full-length hIAPP, as shown in Fig. 6(a). As a result, pore formation is observed when the P/L molar ratio exceeds 7/100. More like AMPs, these fragments tend to translocate through the water pores as shown in Fig. S6. In this way, hIAPP_1–19_ may induce a greater extent of membrane disruption than the full-length peptide at high peptide concentration. In contrast, hIAPP_20–29_ fragments do not bind tightly to the membrane, Fig. S7. These fragments aggregate in the solvent without causing any membrane permeabilization. The movement of some aggregates to another side of the membrane is due to the periodic boundary condition. Because hIAPP_20–29_ fragments are amphiphilic, they can also induce considerable disorder in the membrane, as shown by the induced membrane strain in Fig. 6(a). However, such disorder is not sufficient to porate the membrane due to the native feature of hIAPP_20–29_ lacking of cationic charge. Our results are in agreement with experimental observations^24^,^25^, and can well explain the roles of hIAPP fragments in the membrane disruption: (i) Both of the amphililic fragments in the N-terminal and C-terminal regions have contributions to induce membrane disorder. (ii) The hIAPP_1–19_ fragments carrying cationic charges promote permeable membrane pore formation. (iii) The hIAPP_20–29_ fragments are responsible for amyloid formation. The agreements between our simulations and experiments also indicate the validation of the computational method presented here.

### BSPOTPE molecule inhibits hIAPP-induced membrane damage

A BSPOTPE molecule contains quaternary hydrophobic phenylethene groups and two anionic charges^32^,^33^,^34^ (Figs 1(c) and S3). These characteristics enable it to specifically bind with cationic and amphiphilic *α*-helical hIAPP. Here we simulated 9 to 81 BSPOTPE molecules interacting with 81 hIAPP molecules at the lipid membrane surface.

**Figure 7:**
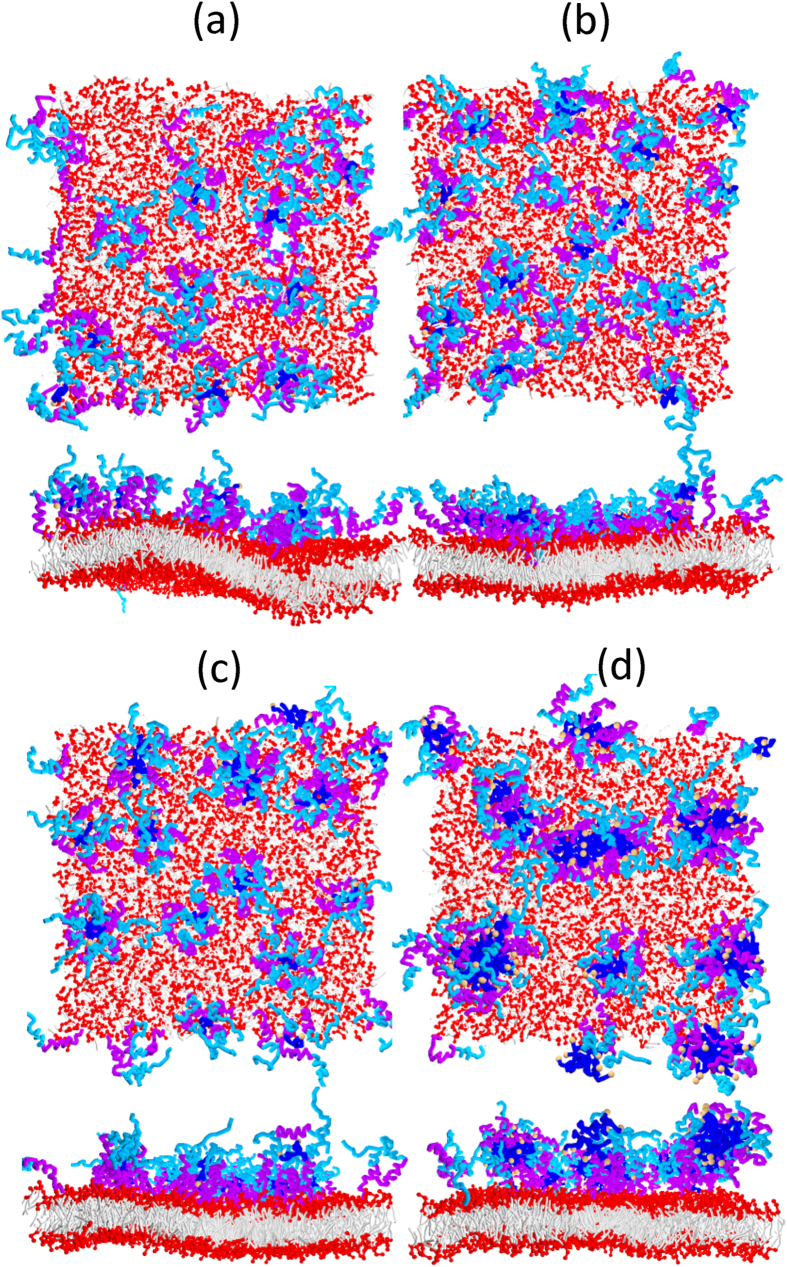
Snapshots of (**a**) 9, (**b**)16, (**c**)25, and (**d**) 81 BSPOTPE molecules interacting with a hIAPP-membrane complex composed of 81 hIAPP molecules and 1600 lipids at a simulation time of 1.144 *μ*s. Both top and intersectional views of the complexes are given.

Figure 7 presents the snapshots of BSPOTPE-hIAPP-membrane complexes containing various numbers of BSPOTPE molecules. At low BSPOTPE/peptide (B/P) molar ratios, for example, B/P < 1/5, one BSPOTPE molecule can bind a couple of hIAPP molecules and form clusters. Non-clustered hIAPP molecules are found binding to the membrane as in the absence of BSPOTPE. When 16 or more BSPOTPE molecules (B/P ≥ 1/5) are added to the system, they can associate with all of the 81 hIAPPs as clusters. Small clusters containing one BSPOTPE molecule and larger clusters containing two or more BSPOTPE molecules are observed. The clusters have micelle-like structure where the phenylethene groups of BSPOTPE and the hydrophobic portions of hIAPP form the hydrophobic cores, while the hydrophilic portions of hIAPP form the water-like shells. At even higher BSPOTPE concentrations [Figs 7(d) and S8], these clusters can further aggregate and lead to prefibril-like structures.

**Figure 8:**
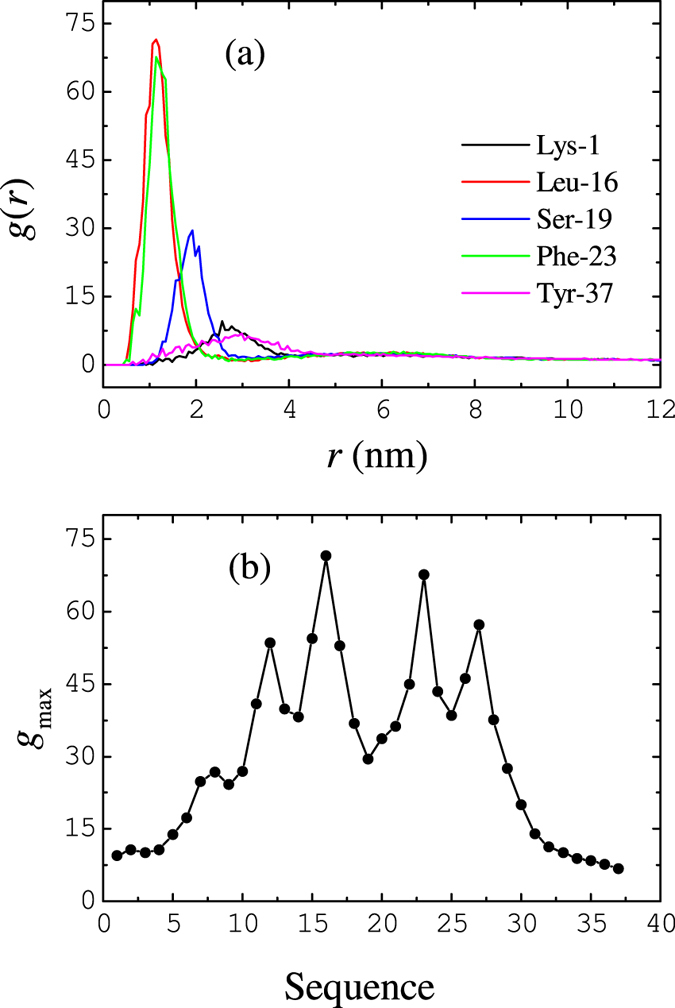
(**a**) Radial distribution functions 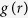 between the center bead of BSPOTPE and typical back-bone beads of hIAPP. (**b**) The maximum value of 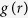 for all of the hIAPP back-bone beads. Data are obtained from a inhibitor-peptide-membrane complex having 9 BSPOTPE, 81 hIAPP , and 1600 lipid molecules.

To identify the binding sites, radian distribution functions (RDFs) 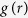 between the center CG bead of BSPOTPE and the back-bone beads of hIAPP are calculated. Several typical RDFs obtained from a system containing 9 BSPOTPE molecules [Fig. 8(a)] show that the hydrophobic residues in the two helical regions of hIAPP locate more closely to BSPOTPE and have high density, while the hydrophilic residues near the two termini only loosely bind to BSPOTPE. The maximum densities abstracted from the RDFs for all of the residues are plotted in Fig. 8(b), which further indicates that the two helices of hIAPP bind tightly to the BSPOTPE molecule. Leu-16 and Phe-23 are the two most confined residues.

**Figure 9:**
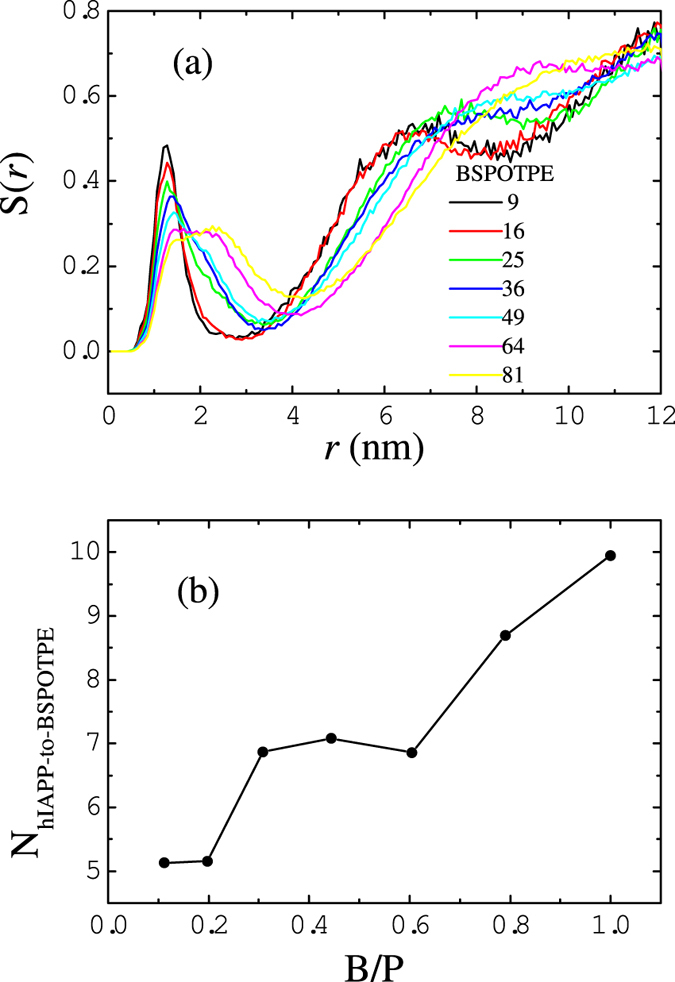
(**a**) Number distribution function 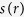 for hIAPP residue Leu-16 around the center bead of BSPOTPE. (**b**) Average number of hIAPP binding to a BSPOTPE molecule as a function of B/P ratio.

In addition to RDFs, we also calculate the number distribution functions of hIAPP residues at a distance *r* around the center bead of BSPOTPE, 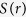. Figure 9(a) presents the 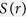 for residue Leu-16 obtained from systems containing various numbers of BSPOTPE . At B/P molar ratios less than 1/5, the narrow distribution of 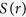 in the first shell (from 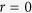 to 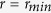; at 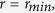 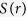 has the first minimum value) indicates that BSPOTPE-hIAPP clusters are well separated. With the addition of BSPOTPE, the association of clusters results in broad and less intense 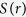 distribution. The sum of 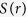 inside the first shell gives rise to the effective average number of hIAPP binding to a BSPOTPE molecule. Figure 9(b) shows the results as a function of the B/P ratio. Three distinct regions are observed: at B/P ≤ 1/5, each BSPOTPE molecule can bind approximately 5 hIAPPs. In this region, the BSPOTPE-hIAPP cluster has a small size and contains only one BSPOTPE molecule. At 1/5 < B/P ≤ 3/5, each BSPOTPE molecule can bind approximately 7 hIAPPs. In this region, some BSPOTPE-hIAPP clusters have a larger size and can contain two BSPOTPE molecules. These combined BSPOTPE molecules share the bound hIAPPs, thus increasing the effective number of bound hIAPPs per BSPOTPE. The number of hIAPPs binding to a BSPOTPE molecule further increases when additional BSPOTPE molecules are present. In this region, the BSPOTPE-hIAPP clusters associate as prefibril-like structures, which promotes even more BSPOTPE molecules to share the bound hIAPPs.

**Figure 10:**
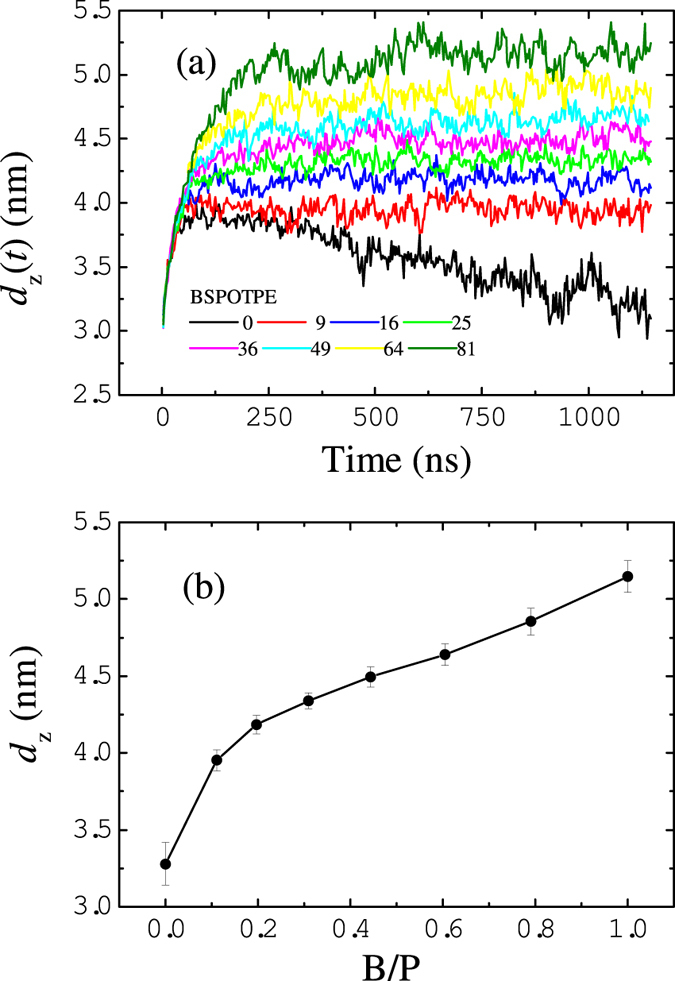
(**a**) Time-dependent distances 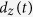 between the center of mass of hIAPP and the membrane center without and with the presence of BSPOTPE. (**b**)Time-averaged distance 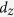 as a function of B/P ratio.

The binding of BSPOTPE to hIAPP lowers the binding affinity of hIAPP to the membrane surface. Here we calculate the particle-averaged distance 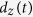 between the center of mass of hIAPP and the membrane center (see the Methods Section). The time-dependent 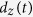 in Fig. 10(a) clearly shows that in the absence of BSPOTPE, hIAPP molecules bind to the lipid membrane with high affinity. The distance keeps decaying because some hIAPP molecules are inserted into and even translocate across the membrane. In contrast, in the presence of BSPOTPE, 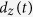 approaches equilibrium after a short relaxation time. The time-averaged distance 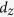 increases with the increasing concentration of BSPOTPE, Fig. 10(b). This suggests that BSPOTPE is competing with the same charged lipids for hIAPP binding, therefore inhibiting the effective binding affinity of hIAPP to the lipid membrane surface.

**Figure 11:**
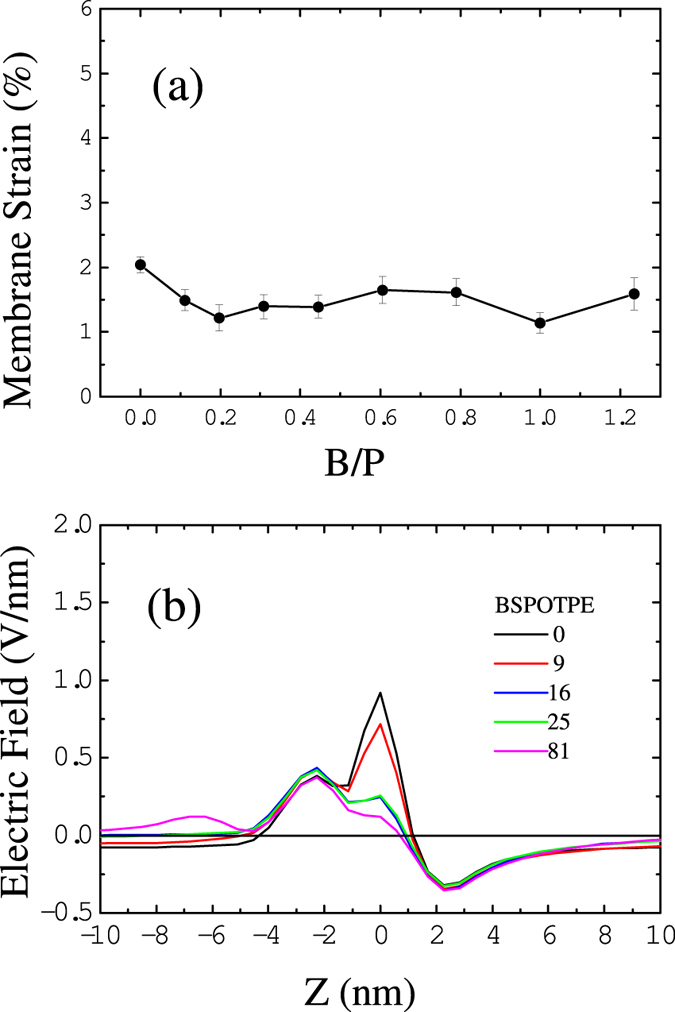
(**a**) Membrane strain and (**b**) Normal component of the electric field as a function of distance Z from the membrane center induced by the binding of 81 hIAPP molecules without and with the presence of BSPOTPE.

The formation of BSPOTPE-hIAPP clusters efficiently prevent hIAPP-induced membrane damage. As shown in Fig. 7, when 16 or more BSPOTPE molecules (B/P ≥ 1/5) approach the hIAPP-membrane complex, which was permeable in the absence of BSPOTPE, water pore disappears. As discussed above, in the presence of BSPOTPE, the hIAPPs only bind weakly to the membrane surface. As a consequence, the bilayer can retain a flat planar configuration. As shown in Fig. 11(a), the area of the membrane can only be stretched by less than 2% at any BSPOTPE concentration simulated here. The presence of BSPOTPE also neutralizes part of the charges of hIAPP and reduce the electric field across the membrane, Fig. 11(b). When 16 or more BSPOTPE are added to the system, the electric field across the membrane is less than 0.4 V/nm, which is not enough to trigger electroporation. These two properties demonstrate that BSPOTPE is able to inhibit hIAPP-induced membrane damage even at a B/P ratio as low as 1/5.

**Figure 12:**
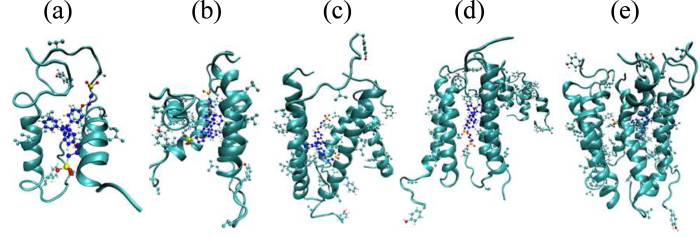
Snapshots of (**a**) 2, (**b**) 4, (**c**) 5, (**d**) 6, and (**e**) 8 hIAPP molecules interacting with a BSPOTPE molecule at a MD simulation time of 20 ns. Only the hydrophobic side-chains of hIAPP are explicitly shown to indicate the binding affinity.

**Figure 13:**
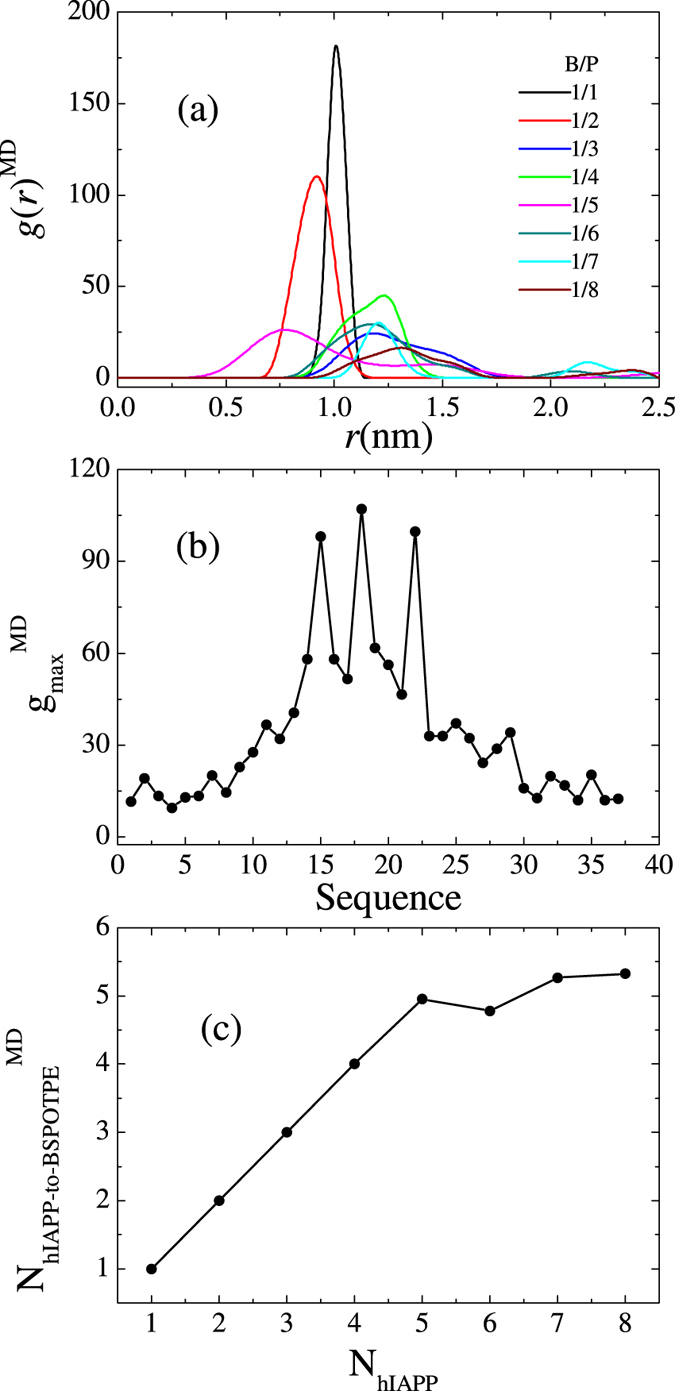
(**a**) Radial distribution functions 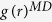 between the center C atom of BSPOTPE and C_*α*_ atom of residue Leu-16 of hIAPP. Data are obtained from MD simulations. (**b**) The maximum value of 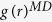 for all of the C_*α*_ atoms of hIAPP. Data are obtained from an inhibitor-peptide complex containing 1 BSPOTPE and 8 molecules. (**c**) Number of hIAPP binding to a BSPOTPE molecule as a function of the number of hIAPP presenting in the system.

To understand why BSPOTPE at a B/P ratio of 1/5 is enough to inhibit hIAPP-induced membrane damage, we also perform extensive atomistic MD simulations of the interaction between one BSPOTPE molecule and multiple hIAPP molecules from a B/P molar ratio of 1/1 to 1/8. The snapshots in Fig. 12 show that when only 5 or less peptides present, all of the hIAPP molecules bind tightly to the BSPOTPE. The peptides adopt extended helical conformations. However, when more than 5 hIAPP peptides present, only 4 to 6 peptides are bound to the BSPOTPE molecule, the excess peptides dissociate from the cluster. The RDFs between 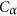 atom of the hydrophobic residue Leu-16 and a center *C* atom of BSPOTPE obtained at various B/P ratios in Fig. 13(a) show that when B/P ≥ 1/5, the RDF has single peak within distance of 1.8 nm; while, when B/P < 1/5, additional peaks at larger distances are observed. RDFs for other residues are also similar to those obtained from DPD simulations. Figure 13(b) represents the maximum values of RDFs for all of the residues simulated at B/P ratio of 1/8, which also demonstrate that the hydrophobic residues in the helical region locate more closely to the inhibitor. Assume that a hIAPP molecule is bound to a BSPOTPE molecule if the distance between Leu-16 and the center of BSPOTPE is within 1.8 nm, then the number of bound hIAPP as a function of the number of hIAPP presenting in the system is counted and given in Fig. 13(c). Consistent with DPD simulations, AAMD simulations prove that a BSPOTPE has ability to adsorb approximately 5 hIAPP molecules. It is worth noting that in ref. 34, the same inhibitor-to-protein ratio of 1/5 was found required to inhibit the insulin fibrillogenesis. It implies that insulin and hIAPP may bind to BSPOTPE through a common mechanism. The consistence between CG simulations, AAMD simulations, and experiments demonstrates the validation of the inhibition mode of BSPOTPE proposed here.

## Discussion

The search for inhibitors to protect *β*-cells from hIAPP-induced toxicity is an important challenge. Revealing the process and mechanism of membrane damage by hIAPP at a molecular level is essential before design and synthesis of potent inhibitors. Our coarse-grained DPD simulations reported here provide a clear picture of the hIAPP-induced membrane poration: Early in the process, hIAPP molecules bind to the surface of the membrane via electrostatic attractions. Then the amphipathic nature of hIAPP drives its hydrophobic face to penetrate into the membrane interior. Above a critical peptide concentration, hIAPP induces multiple toroidal-like pores. The configurations of the hIAPP-membrane complexes from the simulations show that the hydrophobic portions of hIAPP near the N-termini penetrate into the hydrophobic core of the bilayer and disturb the order and organization of the surrounding lipids. Such disordering induces compression on the peptide-rich leaflet and tension on the peptide-free leaflet. Studies on antimicrobial peptides with similar amphiphilicity to hIAPP have suggested that if the local asymmetric tension on the membrane exceeds a critical value of rupture, such as at high peptide concentrations, peptides may associate with lipids, insert into the membrane and form pores. However, we found that hIAPP molecules tend to associate as oligomers and partly shield the hydrophobic portions at high concentrations, thus preventing these molecules from penetrating deeply into the hydrophobic core of the membrane. As a result, these molecules do not cause significant membrane area increase. This finding implies that binding of hIAPP and subsequent membrane disorder and tension are not sufficient to trigger the membrane permeabilization independently. Alternatively, an important feature of hIAPP is that it carries net positive charges. The accumulation of hIAPP at the membrane surface may increase the electric field across the membrane. As a matter of fact, we did observe that when the P/L molar ratio exceeds 5/100, the electric field is high enough to trigger membrane poration. Therefore, the electric field-induced tension compensated the damping of mechanical tension caused by peptide oligomerization at high peptide concentrations. Overall, our simulation demonstrated that peptide-induced membrane tension and electroporation combine together to disrupt the membrane with maximum efficiency.

Different types of inhibitors have been reported, including short peptides derived from the hIAPP sequence^14^,^15^,^16^, native proteins and their fragments^17^,^18^,^19^,^20^,^21^,^22^, and organic compounds^11^,^12^,^13^. These inhibitors were mainly tested for their effects in inhibiting hIAPP fibrillization, and few works have been conducted to determine their effects in protecting membranes from lysing by hIAPP^11^,^13^,^16^. Based on the mechanisms revealed in this work, we propose that a potent inhibitor that can prevent a membrane from being damaged by hIAPP should have capabilities to either screen the hydrophobic portion of hIAPP, neutralize the charges of hIAPP, or both. The organic fluorogen molecule BSPOTPE^32^,^33^,^34^ possesses both of these properties. Our simulations show that this small molecule specifically bind to hIAPP via electrostatic and hydrophobic attractions. The tetraphenylethene sheet structure enable a single BSPOTPE molecule to associate with up to 5 hIAPP molecules as an oligomeric cluster. The cluster has micelle-like structure with a well separated hydrophobic core and hydrophilic shell. Because the hydrophobic portions of hIAPP are well shielded in the micelle-like cluster, the BSPOTPE-hIAPP oligomer has lower ability in penetrating into the bilayer membrane than monomeric hIAPP. The competition between negatively charged BSPOTPE and lipid molecules for hIAPP binding also lowers the binding affinity of hIAPP to the membrane. These two mechanisms work together such that BSPOTPE can effectively inhibit hIAPP-induced membrane damage at B/P molar ratios as low as 1/5.

Geometric structure of an IAPP inhibitor also plays important roles. The flat-sheet structure of BSPOTPE stabilizes the extended *α*-helical conformation of hIAPP that was proposed to be more prone to aggregation^4^. Even though a BSPOTPE molecule only carries two anionic charges, to be well shielded from water, its quaternary hydrophobic phenylethene groups enable it to associate with at least 4 hIAPP molecules via hydrophobic attraction. As shown by both the snapshots and the RDFs obtained from AAMD simulations (Figs 12 and 13), the phenyl rings of BSPOTPE are in contact with the hydrophobic residues of hIAPP, such as leucine, valine, phenylalanine, and tyrosine. At sufficient high hIAPP concentration, the association tendency of hIAPP themselves promotes additional one or two hIAPPs to bind to the cluster. Therefore, A BSPOTPE molecule can accommodate up to 5 hIAPPS. A molecular tweezer, CLR01, which also carries two negative charges, was recently reported as an inhibitor to prevent hIAPP toxicity^13^. However, efficient inhibition of IAPP toxicity requires excess CLR01. The lower accommodation ability of CLR01 may be relevant to the semi-ring structure of CLR01 that stabilized the kinked conformation of IAPP. In one way, the kinked IAPP is less prone to aggregation^13^. In another way, one kinked IAPP might be sufficient to shield the hydrophobic portion of CLR01. Therefore, CLR01 forms a stoichiometric complex with IAPP.

The formation of the BSPOTPE-hIAPP complex potently accelerate the fibrillization in solution. As shown in Fig. 7, at relatively high B/P ratios (B/P > 1/5), two or more BSPOTPE-hIAPP oligomers tend to associate and elongate to prefibril-like structure, which ensures that the hydrophobic part of BSPOTPE can be well screened by touching water. The charge neutralization effect imposed by BSPOTPE also facilitates the hIAPP aggregation. Such a prefibril-like structure enriched in *α*-helical hIAPPs may promote the formation of early intermediate states that allow *β*-sheet formation. This model implies that BSPOTPE is distinct from many other inhibitors that play functions as *β*-sheet breakers. Nevertheless, the prefibril-like aggregation clusters tend to leave off the membrane surface, which actually rescue the membrane from damage.

Helical states of hIAPP have been proposed^35^, as also demonstrated in this work, to play important roles in membrane perturbation and solution phase fiber formation. Thus, a potent inhibitor should interact with the helical region of hIAPP. An example is a synthetic *α*-helical protein mimetic scaffold, IS5, which has been observed to bind directly to the helical region of hIAPP^11^. That scaffold effectively accelerated hIAPP amyloid formation in solution while also inhibiting bilayer catalysis of fibrillogenesis and rescuing hIAPP-induced toxicity in cell culture at an IS5 to peptide ratio of 2:1. Like IS5, as shown in Fig. 8, BSPOTPE also binds directly to the helical regions of hIAPP. More importantly, our simulations indicate that at BSPOTPE to peptide ratios as low as 1:5, BSPOTPE can significantly reduce the toxic effects of hIAPP on the membrane. Our data therefore suggests that BSPOTPE is a good candidate for therapeutic agents that can rescue hIAPP-induced cytotoxicity.

The inhibition mechanism of BSPOTPE against hIAPP-induce membrane damage discussed here is different from most fibrillization inhibitors. Usually, potent fibrillization inhibitors, including charged small molecules^12^, insulin^22^, fragments of hIAPP^14^,^15^, play fibril-breaking functions by stabilizing the native states of hIAPP, or shifting the equilibrium away from an aggregation-prone conformation, and thereby prevent the conformation changes required for protein aggregation^12^. Alternatively, membrane-damage inhibitors, both BSPOTPE and IS5, prevent the adsorption of inhibitor-bound hIAPP oligomeric intermediates onto the lipid membrane, thus decrease the cytotoxicity. This implies that simply inhibiting hIAPP fibrillization might not be able to rescue cells. In contrast, having ability to promote solubilization of inhibitor-bound hIAPP clusters in aqueous phase is more essential for a cell-lysing inhibitor.

In a summary, the results in the current study suggest a novel and highly potential inhibitor, a small organic fluorogen, for inhibiting hIAPP-induced membrane damage. This negatively charged tetraphenylethene molecule specially binds with multiple hIAPP molecules via hydrophobic and electrostatic interactions to form oligomers. The inhibitor-hIAPP oligomers have well separated hydrophilic shells and hydrophobic cores. Such kernelled amphiphilic configuration promote retainment of the inhibitor-bound hIAPP clusters in the water/membrane interface rather than their penetration into the membrane, which finally result in the decrease in cytotoxicity. This study could provide a new ideal for designing inhibitors to rescue hIAPP-induced toxicity in cell culture. We believe that the inhibition effect of BSPOTPE deserves to be investigated experimentally.

## Methods

### DPD simulations

We perform coarse-grained Dissipative Particle Dynamics simulation to investigate the interaction between hIAPP, BSPOTPE, and a model lipid bilayer membrane. In the CG DPD simulation, the elementary unit is a soft bead with each bead representing a fluid volume of several atoms^40^,^41^,^42^. In this work, water is explicitly modeled as a single bead (denoted by W). A lipid molecule is modeled as a polymer connected by harmonic bonds, which consists of four hydrophilic head beads and two tails with six hydrophobic beads. An amino acid residue is represented by one back-bone bead and one or more side-chain beads. A BSPOTPE molecule is represented by a 17-bead polymer with 4 triangle hydrophobic rings and two charged hydrophilic arms. The atomic representation of the DMPC, hIAPP, and BSPOTPE molecules and their corresponding CG models are given in Fig. 1. More detailed CG mapping of the molecules are given in Figs S1–S3 in the Supporting Information. Based on the functional group, the DPD beads are sorted into charged (Q), polar (P), nonpolar (N), and apolar (C) types^43^. Each type is further divided into sublevels based on their hydrogen donor capacities (d), hydrogen acceptor capacities (a), and no hydrogen bond forming capacities (0). The type of the back-bone (B) bead of amino acids depends on the secondary structure of the polypeptide^43^: in a coil or bend structure, B is a strongly polar P_5_ type; in a helical or *β* structure, the polarity is reduced due to the hydrogen bonding between the back-bones; therefore, the bead is a non-polar *N*_0_-type for an *α*-helical structure and a 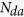-type for a *β*-structure. These beads interact through short-ranged repulsive conservative forces 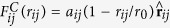, random forces 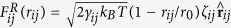, and dissipative forces 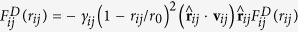 for two beads with a separation 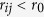
41. Here, vector 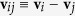 is the velocity difference between particle 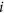 and 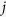. The parameters 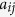 (in units of 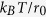) represent the repulsion strengths. The friction coefficients are 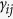 (in units of 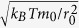). The 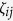 are symmetrically and uniformly distributed random numbers. Optimized DPD force parameters transferrable for both lipids and amino acids and were recently developed by us^37^ were applied here to the hIAPP-membrane system. Detailed information of the CG modeling and force field parameterization can be found in the Supporting Information and ref. 37.

The bond interactions of molecules are described by harmonic potential 
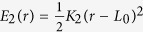
 and angle constraint 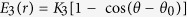
44. Here the equilibrium length 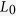, angles 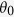, and force constants 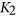 and 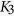 were estimated by performing Boltzmann inversion^45^ of the distribution of bond lengths and angles obtained from AAMD simulations or the Protein Data Bank (PDB). Detailed bond parameters are given in the Supporting Information. For hIAPP, dissociable Morse potential 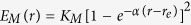 is employed to mimic the hydrogen bonding that stabilizes the *α*-helical structure^46^. Here 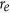 is the equilibrium distance, 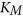 is the the depth of the potential well, and *α* is the width of potential well. For a helical structure, 1–3 Morse bonds between skeleton beads separated by two harmonic bonds and 1–5 bonds between skeleton beads separated by four harmonic bonds are introduced. We set the Morse parameters to 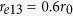, 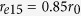, 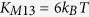, and 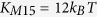 to obtain the “vertical step” of the *α*-helix. The potential width is set to 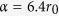


Electrostatic interactions between charged beads are calculated by using the method introduced by Groot^47^. In this method, the charges are distributed on the lattice and the electrostatic potential 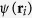 is solved locally on the grid by using a real-space successive over-damped relaxations method 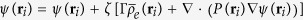
47,48. Here 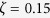 is the analog of a friction factor, 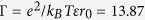 is a coupling constant at room temperature, 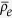 is the averaged local charge density, and 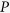 is the polarizability relative to pure water, which is 1 for polar and charged beads, 0.35 for non-polar beads, and 0.025 for apolar beads.

To prepare the initial configuration of the peptide-membrane system, first, a bilayer composed of 1600 lipid molecules is placed in the center of a 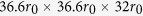-sized box with the head groups on the outside and the alkyl chains inside the membrane. Here 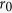 is corresponds to 0.65 nm^42^. These settings ensure that the free bilayer has zero surface tension (see the discussion in the Supporting Information). To simulate typical experimental conditions that can ensure peptide binding, the bilayer consists of 70% DMPC lipids and 30% anionic DMPG lipids. Water beads and counterions are distributed randomly in the space unoccupied by the membrane. The overall bead density 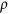 is set to 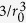. This membrane was relaxed for 50,000 time steps to achieve equilibrium configuration. Then, 16 to 121 hIAPP molecules are uniformly placed on square grids approximately 2 nm above the upper leaflet of the pre-relaxed bilayer membrane. Water and counterions are reloaded by keeping the overall bead density of 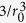. The initial structure of hIAPP is obtained from the PDB (ID: 2L86). To maintain the helical structure, residues 8–18 and 21–29 are constrained by the Morse potential^37^,^46^. To prepare BSPOTPE-hIAPP-membrane complexes, an additional 9 to 81 BSPOTPE molecules are placed uniformly on square grids approximately 1.5 nm above the hIAPP layers. The initial structure of BSPOTPE is obtained from geometry optimization by using the Density Functional Theory with Gaussian 09 package^49^.

DPD simulations are performed in a constant volume and constant temperature (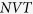) ensemble and periodic boundary conditions by using the velocity-Verlet algorithm^41^. The 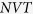 ensemble is chosen to mimic the physical condition of a vesicle-peptide system which has volume confinement. Here the reduced temperature is set to 1, which corresponds to 298 K. The time step is set to 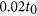, which corresponds to 2.86 ps^42^. Pre-simulations with the positions of the lipids, peptide, and inhibitor fixed are first run for 50,000 time steps to relax the solvent and counterions. Then full simulation for each sample is run for 400,000 time steps (approximately 1.144 *μ*s). At least five independent samples in each condition are simulated to collect data. All of the DPD simulations are performed by using a home-made code package.

### AAMD simulations

The AAMD simulations are performed using GROMACS simulation package^50^ with Gromos 53A6 force field^51^. The systems simulated contain 1, 2, 3, 4, 5, 6, 7 or 8 hIAPP molecules,1 BSPOTPE molecule, 9368, 9170, 8991, 8789, 8605, 8400, 8220 or 8035 water molecules, and 1, 4, 7, 10, 13, 16, 19 or 22 chloride counterions to neutralize the system. The BSPOTPE molecule with hIAPP molecules surrounding it is placed in the center of the rectangular box of dimensions 5.0 nm × 5.0 nm × 10.0 nm. All of the simulations are performed under the NVT ensemble (300 K) with coupling constant 0.1 ps for temperature of Nosé Hoover thermostat^52^. The cutoff method is used for van der Waals interaction with a radius of 1.2 nm. Particle Mesh Ewald method is used to calculate the long-range electrostatic potential^53^,^54^, where the radius for the short range is set at 1.2 nm. Neighbor lists are updated every 5 steps. LINCS is used to constrain all bonds^55^. A 50000-step energy minimization is first performed using the steepest descent method. The solvent is then relaxed by applying a restrain potential on heavy atoms of hIAPP and BSPOTPE for 100 ps. Under the native condition, a 20 ns simulation is run for production. A RDF is obtained from 100 samples in the final 2 ns.

### Calculation of membrane leakage

The membrane leakage is estimated by counting the number of water beads in the membrane pore. To do the counting, we first divide the simulation box into cells, with each cell having size 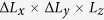. Here we set 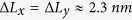. This setting is to ensure that the membrane patch in each cell is small enough to ignore its local curvature and large enough to involve a few lipids. Then we calculate the position of the center of mass of lipids 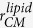 in each cell. If the distance between a water bead and 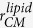 in the *Z* direction is less than half of the membrane thickness 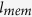 (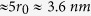), this water is considered to be inside the membrane pore. If no lipid is found in a cell, there must be a pore formed therein. The amount of water trapped in the pore in that cell is estimated to be 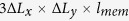.

### Calculation of membrane strain

The percentage change in the membrane area induced by hIAPP binding is calculated by 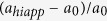. Here 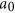 is the area of a membrane with zero surface tension and in the absence of bound peptides. The area of a peptide-bound membrane, 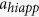, is estimated from the average taken for 20 ns after the peptides fully bind on the surface of the membrane but before pore formation. To measure 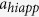, we also divide the simulation box into cells and calculate the center of mass of lipids 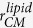 in each cell as in the calculation of membrane leakage. Then all of the 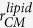 are connected by triangle network. The area of the membrane is approximately equal to the sum of the area of all of the triangles.

### Calculation of binding affinity of hIAPP to lipid membrane

The binding affinity of hIAPP to the lipid membrane can be measured by calculating the particle-averaged normal distance between the center of mass of hIAPP (or the position of individual back-bone bead) and the membrane center. Similar to the method of counting membrane leakage, here, we also divide the simulation box into cells. The distance in the Z-direction between hIAPP and lipids in all of the cells is then calculated and averaged.

## Additional Information

**How to cite this article**: Li, X. *et al*. Mechanism of Inhibition of Human Islet Amyloid Polypeptide-Induced Membrane Damage by a Small Organic Fluorogen. *Sci. Rep*. **6**, 21614; doi: 10.1038/srep21614 (2016).

## Supplementary Material

Supplementary Information

## References

[b1] Lorenzo A., Razzaboni B., Weir G. C. & Yankner B. A. Pancreatic-sslet cell toxicity of amylin associated with type-2 diabetes-mellitus. Nature 368, 756–760 (1994). 8152488 10.1038/368756a0

[b2] Kahn S. E., Andrikopoulos S. & Verchere C. B. Islet amyloid: a long-recognized but underappreciated pathological feature of type 2 diabetes. Diabetes 48, 241–253 (1999). 10334297 10.2337/diabetes.48.2.241

[b3] Janson J., Ashley R. H., Harrison D., McIntyre S. & Butler P. C. The mechanism of islet amyloid polypeptide toxicity is membrane disruption by intermediate-sized toxic amyloid particles. Diabetes 48, 491–498 (1999). 10078548 10.2337/diabetes.48.3.491

[b4] Knight J. D., Hebda J. A. & Miranker A. D. Conserved and cooperative assembly of membrane-bound alpha-helical states of islet amyloid polypeptide. Biochemistry 45, 9496–9508 (2006). 16878984 10.1021/bi060579z

[b5] Last N. B., Rhoades E. & Miranker A. D. Islet amyloid polypeptide demonstrates a persistent capacity to disrupt membrane integrity. Proc. Natl. Acad. Sci. USA 108, 9460–9465 (2011). 21606325 10.1073/pnas.1102356108 PMC3111278

[b6] Quist A. . Amyloid ion channels: a common structural link for protein-misfolding disease. Proc. Natl. Acad. Sci. USA 102, 10427–10432 (2005). 16020533 10.1073/pnas.0502066102 PMC1180768

[b7] Cao P. . Islet amyloid polypeptide toxicity and membrane interactions. Proc. Natl. Acad. Sci. USA 110, 19279–19284 (2013). 24218607 10.1073/pnas.1305517110 PMC3845181

[b8] Green J. D. . Atomic force microscopy reveals defects within mica supported lipid bilayers induced by the amyloidogenic human amylin peptide. J. Mol. Biol. 342, 877–887 (2004). 15342243 10.1016/j.jmb.2004.07.052

[b9] Engel M. F. . Membrane damage by human islet amyloid polypeptide through fbril growth at the membrane. Proc. Natl. Acad. Sci. USA 105, 6033–6038 (2008). 18408164 10.1073/pnas.0708354105 PMC2329711

[b10] Milanesi L. . Direct three-dimensional visualization of membrane disruption by amyloid fibrils. Proc. Natl. Acad. Sci. USA 109, 20455–20460 (2012). 23184970 10.1073/pnas.1206325109 PMC3528594

[b11] Hebda J. A., Saraogi I., Magzoub M., Hamilton A. D. & Miranker A. D. A peptidomimetic approach to targeting pre-amyloidogenic states in type ii diabetes. Chem. Biol. 16, 943–950 (2009). 19778722 10.1016/j.chembiol.2009.08.013 PMC3341621

[b12] Young L. M. . Screening and classifying small-molecule inhibitors of amyloid formation using ion mobility spectrometry-mass spectrometry. Nat. Chem. 7, 73–81 (2015). 25515893 10.1038/nchem.2129 PMC4280571

[b13] Lopes D. H. . Molecular tweezers inhibit islet amyloid polypeptide assembly and toxicity by a new mechanism. ACS Chem. Biol. 10, 15551569 (2015). 10.1021/acschembio.5b00146 25844890

[b14] Scrocchi L. A. . Design of peptide-based inhibitors of human islet amyloid polypeptide fibrillogenesis. J. Mol. Biol. 318, 697706 (2002). 10.1016/S0022-2836(02)00164-X 12054816

[b15] Mishra A. . Conformationally restricted short peptides inhibit human islet amyloid polypeptide (hiapp) fibrillization. Chem. Commun. 49, 2688–2690 (2013). 10.1039/c3cc38982k PMC3684849 23435449

[b16] Wang L., Lei L., Li Y., Wang L. & Li F. A hiapp-derived all-d-amino-acid inhibits hiapp fibrillation efficiently at membrane surface by targeting alpha-helical oligomeric intermediates. FEBS Lett. 588, 884–891 (2014). 24561193 10.1016/j.febslet.2014.02.020

[b17] Larson J. L. & Miranker A. D. The mechanism of insulin action on islet amyloid polypeptide fiber formation. J. Mol. Biol. 335, 221–231 (2004). 14659752 10.1016/j.jmb.2003.10.045

[b18] Yan L. M., Tatarek-Nossol M., Velkova A., Kazantzis A. & Kapurniotu A. Design of a mimic of nonamyloidogenic and bioactive human islet amyloid polypeptide (iapp) as nanomolar affinity inhibitor of iapp cytotoxic fibrillogenesis. Proc. Natl. Acad. Sci. USA 103, 2046–2051 (2006). 16467158 10.1073/pnas.0507471103 PMC1413694

[b19] Abedini A., Meng F. & Raleigh D. P. A single-point mutation converts the highly amyloidogenic human islet amyloid polypeptide into a potent fibrillization inhibitor. J. Am. Chem. Soc. 129, 11300–11301 (2007). 17722920 10.1021/ja072157y

[b20] Knight J. D., Williamson J. A. & Miranker A. D. Interaction of membrane-bound islet amyloid polypeptide with soluble and crystalline insulin. Protein Sci. 17, 1850–1856 (2008). 18765820 10.1110/ps.036350.108 PMC2548354

[b21] Meng F., Raleigh D. P. & Abedini A. Combination of kinetically selected inhibitors in trans leads to highly effective inhibition of amyloid formation. J. Am. Chem. Soc. 132, 14340–14342 (2010). 20873820 10.1021/ja1046186 PMC3199963

[b22] Susa A. C. . Defining the molecular basis of amyloid inhibitors: human islet amyloid polypeptide-insulin interactions. J. Am. Chem. Soc. 136, 12912–12919 (2014). 25144879 10.1021/ja504031d PMC4183647

[b23] Last N. B. & Miranker A. D. Common mechanism unites membrane poration by amyloid and antimicrobial peptides. Proc. Natl. Acad. Sci. USA 110, 6382–6387 (2013). 23576726 10.1073/pnas.1219059110 PMC3631635

[b24] Brender J. R. . Amyloid fiber formation and membrane disruption are separate processes localized in two distinct regions of iapp, the type-2-diabetes-related peptide. J. Am. Chem. Soc. 130, 6424–6429 (2008). 18444645 10.1021/ja710484d PMC4163023

[b25] Brender J. R. . Membrane disordering is not sufficient for membrane permeabilization by islet amyloid polypeptide: studies of iapp(20–29) fragments. Phys. Chem. Chem. Phys. 15, 8908–8915 (2013). 23493863 10.1039/c3cp44696d PMC3663912

[b26] Buchanan L. E. . Mechanism of iapp amyloid fibril formation involves an intermediate with a transient beta-sheet. Proc. Natl. Acad. Sci. USA 110, 19285–19290 (2013). 24218609 10.1073/pnas.1314481110 PMC3845187

[b27] Dupuis N. F., Wu C., Shea J. E. & Bowers M. T. The amyloid formation mechanism in human iapp: dimers have beta-strand monomer-monomer interfaces. J. Am. Chem. Soc. 133, 7240–7243 (2011). 21517093 10.1021/ja1081537 PMC3093713

[b28] Qiao Q., Bowman G. R. & Huang X. Dynamics of an intrinsically disordered protein reveal metastable conformations that potentially seed aggregation. J. Am. Chem. Soc. 135, 16092–16101 (2013). 24021023 10.1021/ja403147m

[b29] Zhang Y., Luo Y., Deng Y., Mu Y. & Wei G. Lipid interaction and membrane perturbation of human islet amyloid polypeptide monomer and dimer by molecular dynamics simulations. PLoS One 7, e38191 (2012). 22693597 10.1371/journal.pone.0038191 PMC3364971

[b30] Zhao J. . Probing ion channel activity of human islet amyloid polypeptide (amylin). Biochim. Biophys. Acta 1818, 3121–3130 (2012). 22935354 10.1016/j.bbamem.2012.08.012 PMC3455117

[b31] Pannuzzo M., Raudino A., Milardi D., La Rosa C. & Karttunen M. Alpha-helical structures drive early stages of self-assembly of amyloidogenic amyloid polypeptide aggregate formation in membranes. Sci. Rep. 3, 2781 (2013). 24071712 10.1038/srep02781 PMC3784961

[b32] Tong H. . Protein detection and quantitation by tetraphenylethene-based fluorescent probes with aggregation-induced emission characteristics. J. Phys. Chem. B 111, 11817–11823 (2007). 17877385 10.1021/jp073147m

[b33] Hong Y. . Quantitation, visualization, and monitoring of conformational transitions of human serum albumin by a tetraphenylethene derivative with aggregation-induced emission characteristics. Anal. Chem. 82, 7035–7043 (2010). 20704392 10.1021/ac1018028

[b34] Hong Y. . Monitoring and inhibition of insulin fibrillation by a small organic fluorogen with aggregation-induced emission characteristics. J. Am. Chem. Soc. 134, 1680–1689 (2012). 22191699 10.1021/ja208720a

[b35] Hebda J. A. & Miranker A. D. The interplay of catalysis and toxicity by amyloid intermediates on lipid bilayers: insights from type ii diabetes. Annu. Rev. Biophys. 38, 125–152 (2009). 19416063 10.1146/annurev.biophys.050708.133622

[b36] Zasloff M. Antimicrobial peptides of multicellular organisms. Nature 415, 389–395 (2002). 11807545 10.1038/415389a

[b37] Chen L., Li X., Gao L. & Fang W. Theoretical insight into the relationship between the structures of antimicrobial peptides and their actions on bacterial membranes. J. Phys. Chem. B 119, 850–860 (2015). 25062757 10.1021/jp505497k

[b38] Jean-Francois F., Elezgaray J., Berson P., Vacher P. & Dufourc E. J. Pore formation induced by an antimicrobial peptide: electrostatic effects. Biophys. J. 95, 5748–5756 (2008). 18820233 10.1529/biophysj.108.136655 PMC2599824

[b39] Tieleman D. P., Leontiadou H., Mark A. E. & Marrink S. J. Simulation of pore formation in lipid bilayers by mechanical stress and electric fields. J. Am. Chem. Soc. 125, 6382–6383 (2003). 12785774 10.1021/ja029504i

[b40] Espanol P. & Warren P. Statistical mechanics of dissipative particle dynamics. Eur. Phys. Lett. 30, 191 (1995).

[b41] Groot R. D. & Warren P. B. Dissipative particle dynamics: bridging the gap between atomistic and mesoscopic simulation. J. Chem. Phys. 107, 4423 (1997).

[b42] Groot R. & Rabone K. Mesoscopic simulation of cell membrane damage, morphology change and rupture by nonionic surfactants. Biophys. J. 81, 725–736 (2001). 11463621 10.1016/S0006-3495(01)75737-2 PMC1301549

[b43] Monticelli L. . The MARTINI coarse-grained force field: extension to proteins. J. Chem. Theory Comput. 4, 819–834 (2008). 26621095 10.1021/ct700324x

[b44] Shillcock J. C. & Lipowsky R. Equilibrium structure and lateral stress distribution of amphiphilic bilayers from dissipative particle dynamics simulations. J. Chem. Phys. 117, 5048–5061 (2002).

[b45] Milano G. & Muller-Plathe F. Mapping atomistic simulations to mesoscopic models: a systematic coarse-graining procedure for vinyl polymer chains. J. Phys. Chem. B 109, 18609–18619 (2008). 16853395 10.1021/jp0523571

[b46] Vishnyakov A., Talaga D. S. & Neimark A. V. DPD simulation of protein conformations: From α-helices to *β*-structures. J. Phys. Chem. Lett. 3, 3081–3087 (2012). 26296009 10.1021/jz301277b

[b47] Groot R. D. Electrostatic interactions in dissipative particle dynamics simulation of polyelectrolytes and anionic surfactants. J. Chem. Phys. 118, 11265 (2003).

[b48] Gao L. & Fang W. Communications: Self-energy and corresponding virial contribution of electrostatic interactions in dissipative particle dynamics: Simulations of cationic lipid bilayers. J. Chem. Phys. 132, 031102 (2010). 20095720 10.1063/1.3297889

[b49] Frisch, e. a. M. J. *Gaussian 09, Revision D.01* Gaussian, Inc., Wallingford CT, (2009).

[b50] Hess B., Kutzner C., van der Spoel D. & Lindahl E. Gromacs 4: Algorithms for highly efficient, load-balanced, and scalable molecular simulation. J. Chem. Theo. Comp. 4, 435–447 (2008). 10.1021/ct700301q 26620784

[b51] Oostenbrink C., Villa A., Mark A. E. & van Gunsteren W. F. A biomolecular force field based on the free enthalpy of hydration and solvation: the gromos force-field parameter sets 53a5 and 53a6. J. Comput. Chem. 25, 1656–1676 (2004). 15264259 10.1002/jcc.20090

[b52] Hoover W. G. Nosé Hoover nonequilibrium dynamics and statistical mechanics. Mol. Simulat. 33, 13–19 (2007).

[b53] Essmann U. . A Smooth Particle Mesh Ewald Method. J. Chem. Phys. 103, 8577–8593 (1995).

[b54] Darden T., York D. & Pedersen L. Particle Mesh Ewald-an N. Log(N) Method for Ewald Sums In Large Systems. J. Chem. Phys. 98, 10089–10092 (1993).

[b55] Hess B., Bekker H., Berendsen H. J. C. & Fraaije J. G. E. M. LINCS: A linear constraint solver for molecular simulations. J. Comput. Chem. 18, 1463–1472 (1997).

