# Supplementary material for: Mechanism of Inhibition of Human Islet Amyloid Polypeptide-Induced Membrane Damage by a Small Organic Fluorogen
Source: Sci Rep. 2016 Feb 18;6:21614. doi: 10.1038/srep21614 (PMC4757883; doi:10.1038/srep21614)
Supplement: Supplementary Information [file srep21614-s1.pdf]

Mechanism of Inhibition of Human Islet Amyloid Polypeptide-Induced Membrane Damage by a Small Organic Fluorogen - Supporting Information

Xiaoxu Li[1], Mingwei Wan[1], Lianghui Gao[1,*], and Weihai Fang[1]

[1]Key Laboratory of Theoretical and Computational Photochemistry, Ministry of Education, College of Chemistry, Beijing Normal University, Beijing 100875, China
[*]lhgao@bnu.edu.cn

ABSTRACT

Human islet amyloid polypeptide (hIAPP) is believed to be responsible for the death of insulin-producing β-cells. However, the mechanism of membrane damage at the molecular level has not been fully elucidated. In this article, we employ coarse-grained dissipative particle dynamics simulations to study the interactions between a lipid bilayer membrane composed of 70% zwitterionic lipids and 30% anionic lipids and hIAPPs with α-helical structures. We demonstrated that the key factor controlling pore formation is the combination of peptide charge-induced electroporation and peptide hydrophobicity-induced lipid disordoring and membrane thinning. According to these mechanisms, we suggest that a water-miscible tetraphenylethene BSPOTPE is a potent inhibitor to rescue hIAPP-induced cytotoxicity. Our simulations predict that BSPOTPE molecules can bind directly to the helical regions of hIAPP and form oligomers with separated hydrophobic cores and hydrophilic shells. The micelle-like hIAPP-BSPOTPE clusters tend to be retained in the water/membrane interface and aggregate therein rather than penetrate into the membrane. Electrostatic attraction between BSPOTPE and hIAPP also reduces the extent of hIAPP binding to the anionic lipid bilayer. These two modes work together and efficiently prevent membrane poration.

CG Mapping Model

In the coarse grained DPD model, based on the functional group, approximately three to four heavy atoms together with their attached hydrogens are mapped to a single bead. Accordingly, a DMPC lipid molecule is modeled as a polymer connected by harmonic bonds, which consists of four hydrophilic head beads and two tails. An amino acid residue is represented by one backbone bead and one or more side-chain beads. A BSPOTPE molecule is model as a four-ring hydrophobic sheet with two charged arms. The atomic representations and corresponding CG models of these molecules are are given in Figs. S1 to S3. The beads are labeled in different colors to represent their types.

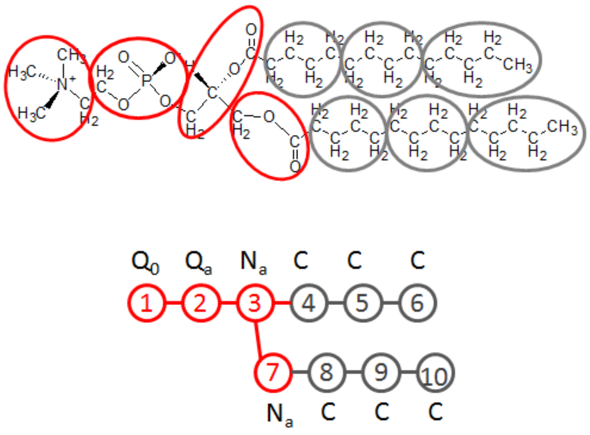

Figure S1. Atomic representation of DMPC and its corresponding CG model.

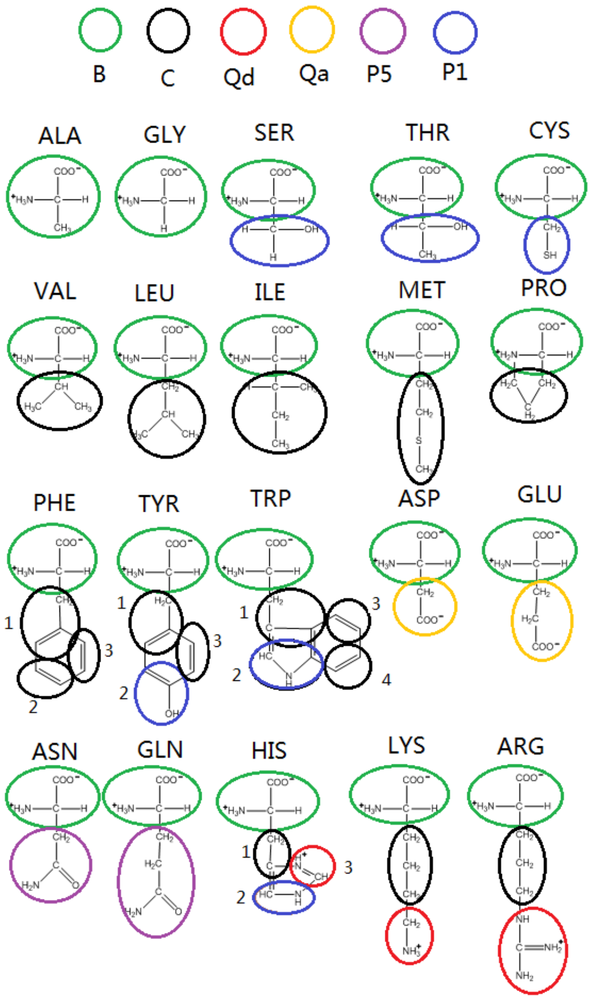

Figure S2. Atomic representation of protein amino acids and their corresponding CG models.

Force Field Parameterization

In DPD simulations, the repulsion parameter a_{ij} are usually optimized to reproduce the compressibility of the system.[1,2] For a pure water system, when the bead density $\rho > 2$, the choice of water-water repulsion $a_{WW} \geq 15$ can accurately reproduce the water compressibility at reduced temperature $T^* = 1$.[1,2] At $\rho = 3$, a_{WW} is usually set to 78 in the three-to-one mapping model. The repulsion between beads of the same type is equal to a_{WW}. The other force parameters a_{ij} between beads of different types are obtained from the relationship between the mutual solubility of polymers in water,[1,2] which is expressed by the Flory-Huggins χ-parameter and the excess repulsion $\Delta a_{ij} = a_{ij} - a_{WW}$,

$$\chi = 0.231\Delta a_{ij}. \quad (1)$$

The Flory-Huggins χ-parameter can be obtained from experiments or all-atom simulations. Because not all of the Flory-Huggins χ-parameters can be obtained experimentally, we have performed all atomistic simulations of the molecular fragments with different CG types to derive the χ-parameters using the Blends modules in the Materials Studio package.[3] In the simulations, a broken carbon-bond is linked by a hydrogen atom, while other types of broken bonds are linked by methyl groups. Polymer consistent force field (PCFF) was used in the simulations, which is intended for application to polymers and organic materials.[3] The estimated χ values for uncharged beads of types W, P_5, P_1, N_a, and C, and the corresponding DPD force parameters a_{ij} are given in Table S1. The χ values of 10.88 and 0.53 for W-C, W-Na pairs are relatively higher than the experimental data, which are approximately 6.0 (or 9.3) and 0.3.[2] One possible reason for the difference is that the bead in the simulation is a fragmental piece rather a whole molecule. Here we use the same force parameters given by Groot and Rabone[2] for W-C pair. We note that in the DPD lipid model in Ref.,[2] the two ethyleneoside sites were considered as one

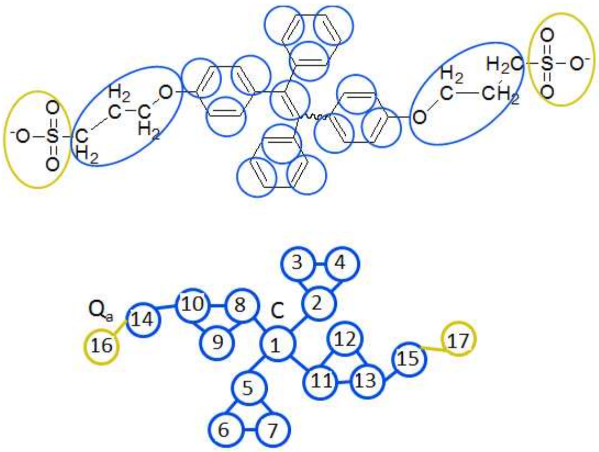

Figure S3. Atomic representation of BSPOTPE and its corresponding CG model.

Table S1. Flory-Huggins χ-parameters and corresponding DPD force parameters a_{ij} (k_BT/r_0) for beads with type W, P_5, P_1, N_a, and C.

χ/a_{ij}	W	P_5	P_1	N_a	C
W		-1.30/72.4	0.51/80.2	0.53/80.4	10.88/125
P_5			-0.25/76.9	2.16/87.4	7.32/109.7
P_1				0.36/79.6	3.23/92.0
N_a					1.47/84.4

N_a-type CG bead. In our mapping scheme, there are two N_a-type beads, thus the hydrophilicity of the surfactant in these two models is different. To reproduce the structure and elastic properties of the lipid bilayer membrane, we carefully reduce the force parameters between the lipid head group and water a_{WQ_0} and a_{WQ_a} from 75.8 to 72 and a_{WN_a} from 86.7 to 83. By these parameters (as well as the bond parameters discussed next), DMPC membrane at tensionless state has thickness of 3.72 nm, and area per lipid at 0.68 nm^2. These structural properties are in good agreement with experimental measurements.[4] We also found that the membrane simulated here have area compressibility of 130 dyn/cm and bending rigidity $\approx 0.33\times10^{-19}$ J. The membrane ruptures when the its area is stretched by less than 10%, and the rupture tension is approximately 10 mN/m. These mechanical properties are also comparable to the experimental data.[5]

Next, we expand the force parameters to amino acids. Table S1 shows that the most polar P_5-type bead (including amide group) has strong hydrophilicity. The P_1-type bead (including hydroxyl or hydrosulfide groups) is less polar and its hydrophilicity is similar to that of the N_a-type bead. These bead-bead interactions can be sorted into super attractive, attractive, almost attractive, intermediate, almost repulsive, repulsive, and super repulsive levels.[6–9] For the charged Q-type beads, we assume that their polarities are similar to those of P_5-type beads. The non-polar N_0-type bead has no hydrogen bonding ability, thus it has a lower hydrophilicity than the N_a-type bead. The N_{da}-type bead has a similar hydrophilicity to the N_a-type bead. Based on these properties, suitable force parameters at the proper interactions levels are extracted from Table 1 and assigned to all of the CG beads. The final force parameters for the 10 types of beads discussed in this article are given in Table S2.

The equilibrium CG bond lengths and angles and the respective force constants of lipids are obtained by fitting the bond distributions derived from AAMD simulations.[10] First, we simulate 16 DMPC lipids in a box containing 1600 water molecules using Amber force fields. In this concentration, the DMPC lipids do not form ordered structure. We then calculate the bond and angle distributions of the center of masses of the CG beads. The distributions are fitted by Gaussian functions[10]

$$P(\theta) = \frac{A}{w\sqrt{\pi/2}}\exp^{-2(\theta-\theta_c)^2/w^2}. \quad (2)$$

Here, the structure parameter θ can be a bond or an angle. The fitting parameter θ_c is the distribution center, A is the area, and w is the width, which is related to the force constant by $K_2\ (or\ K_3) = 4k_BT/w^2$. For example, fits of the C-C bond and C-C-C angle distributions of the DPPC molecules give $L_0 \approx 0.47nm \approx 0.72r_0$, $K_2 \approx 512k_BT/r_0^2$, $\theta_0 \approx 174^\circ$, and $K_3 \approx 10k_BT/r_0^2$. For

Table S2. DPD force parameters a_{ij} (k_BT/r_0).

a_{ij}	W	Q_0	Q_d	Q_a	N_a	C	P_5	P_1	N_0	N_{da}
W	78	72	72	72	83	104	72	79.3	86.7	83
Q_0		86.7	79.3	79.3	83	104	78	83	86.7	83
Q_d			78	72	78	104	72	78	86.7	79.3
Q_a				78	83	104	72	78	86.7	78
N_a					78	92	86.7	79.3	86.7	79.3
C						78	104	92	92	92
P_5							72	72	86.7	78
P_1								78	83	79.3
N_0									78	86.7
N_{da}										78

Table S3. Equilibrium bond lengths, angles,and force parameters for DPPC lipid.

bond	L_0 (r_0)	K_2 (k_BT/r_0^2)	angle	θ_0 (degree)	K_3 (k_BT)
1-2	0.56	512	2-3-4	180	10
2-3	0.56	512	2-3-7	90	10
3-7	0.42	512	3-4-5	180	10
3-4	0.72	512	4-5-6	180	10
4-5	0.72	512	5-6-7	180	10
5-6	0.72	512	7-8-9	180	10
7-8	0.72	512	8-9-10	180	10
8-9	0.72	512			
9-10	0.72	512			

lipids in a bilayer, the hydrocarbon tails are more compacted, so we choose θ_0 to be 180° for C-C-C angles. All of the bond parameters for DMPC are given in Table S3. For BSPOTPE, the CG bond lengths are estimated based on the configuration obtained from DFT optimization, Table S4. The bond force constants are also set to $K_2 \approx 512 k_BT/r_0^2$. For polypeptides, only the harmonic bond potential is considered. The bond lengths can be estimated from the distributions derived from the PDB. We use the data obtained from up to 1000 proteins; these are also given in Table S5 . The corresponding bond force constants are all set to $K_2 \approx 512 k_BT/r_0^2$.

Table S4. Equilibrium bond lengths, and force parameters for BSPOTPE .

bond	L_0 (r_0)	K_2 (k_BT/r_0^2)
1-2	0.42	512
1-5	0.42	512
1-8	0.42	512
1-11	0.42	512
2-3	0.46	512
2-4	0.46	512
3-4	0.46	512
5-6	0.46	512
5-7	0.46	512
6-7	0.46	512
8-9	0.46	512
8-10	0.46	512
9-10	0.46	512
10-14	0.64	512
11-12	0.46	512
11-13	0.46	512
12-13	0.46	512
13-15	0.64	512
14-16	0.55	512
15-17	0.55	512

Table S5. Equilibrium bond lengths for amino acids. B stands for backbone bead and S stands for side-chain bead.

amino acid	L_0 (r_0)	amino acid	L_0 (r_0)
backbone L_{BB}	0.54	His L_{BS1}	0.49
Leu L_{BS}	0.50	His L_{S1S2}	0.36
Ile L_{BS}	0.48	His L_{S1S3}	0.36
Val L_{BS}	0.40	His L_{S2S3}	0.27
Pro L_{BS}	0.46	Phe L_{BS1}	0.48
Met L_{BS}	0.61	Phe L_{S1S2}	0.36
Cys L_{BS}	0.48	Phe L_{S1S2}	0.41
Ser L_{BS}	0.38	Phe L_{S1S2}	0.33
Thr L_{BS}	0.40	Tyr L_{BS1}	0.49
Asn L_{BS}	0.49	Tyr L_{S1S2}	0.36
Gln L_{BS}	0.61	Tyr L_{S1S3}	0.48
Asp L_{BS}	0.49	Tyr L_{S2S3}	0.35
Glu L_{BS}	0.61	Trp L_{BS1}	0.46
Arg L_{BS}	0.49	Trp L_{S1S2}	0.32
Arg L_{SS}	0.50	Trp L_{S1S3}	0.45
Lys L_{BS}	0.50	Trp L_{S2S4}	0.46
Lys L_{SS}	0.43	Trp L_{S3S4}	0.33
Cys-Cys L_{SS}	0.60		

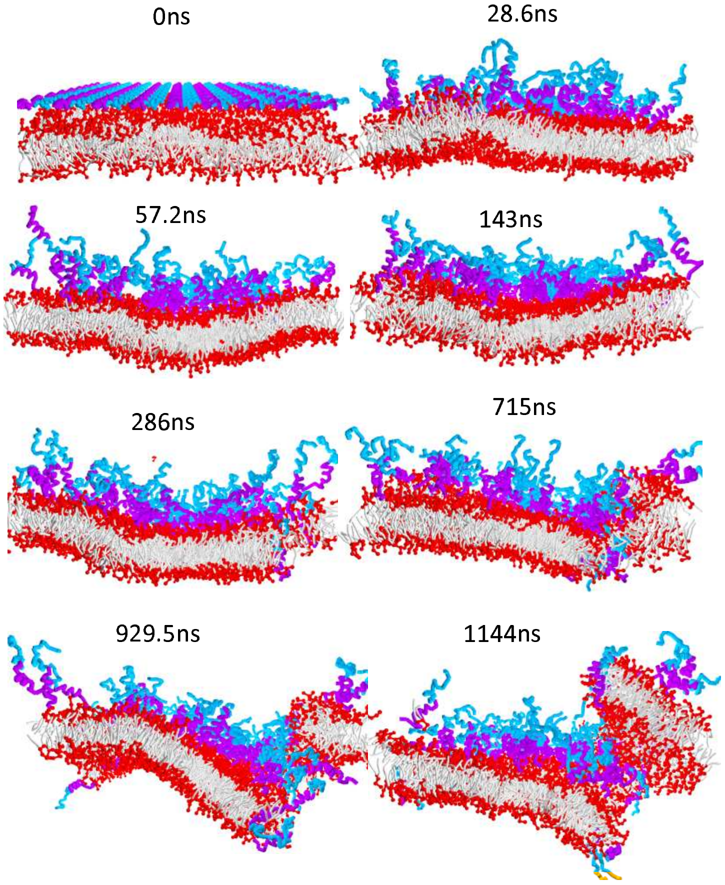

Figure S4. Time evalutional snapshots of 81 hIAPP molecules binding to a bilayer membrane composed of 1600 lipids.

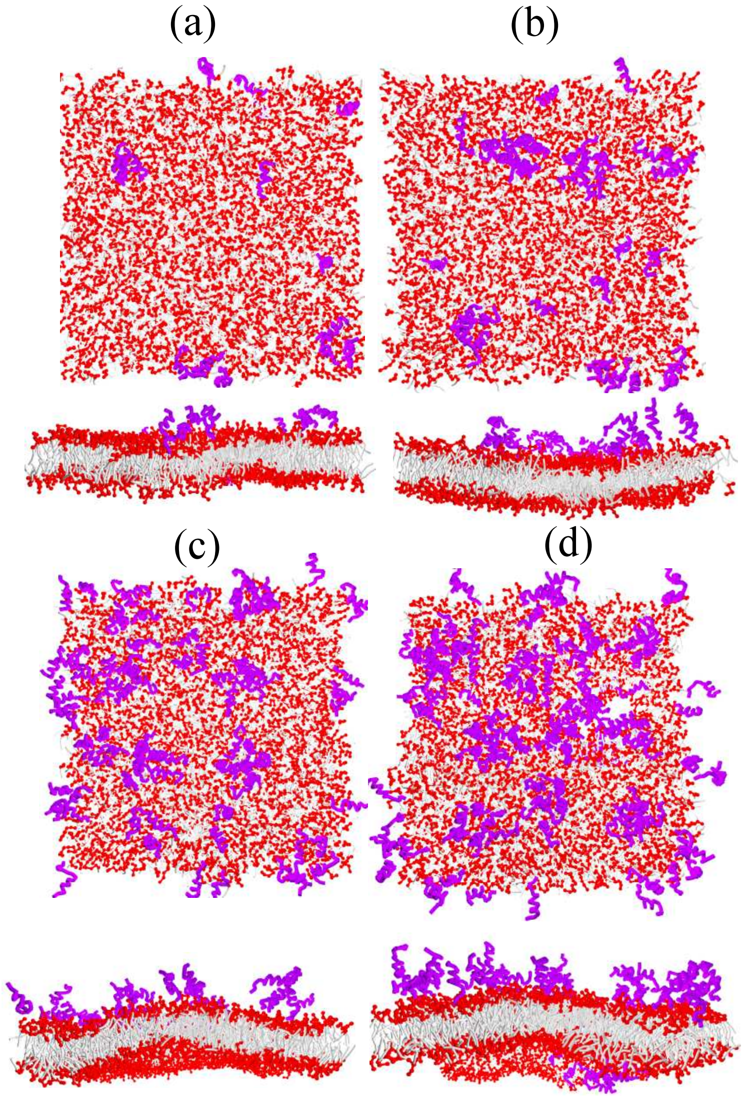

Figure S5. Snapshots of (a) 16, (b) 36, (c) 81, (d) 121 $hIAPP_{1-19}$ fragments interacting with a bilayer membrane composed of 1600 lipids at a simulation time of 1.144 μs.

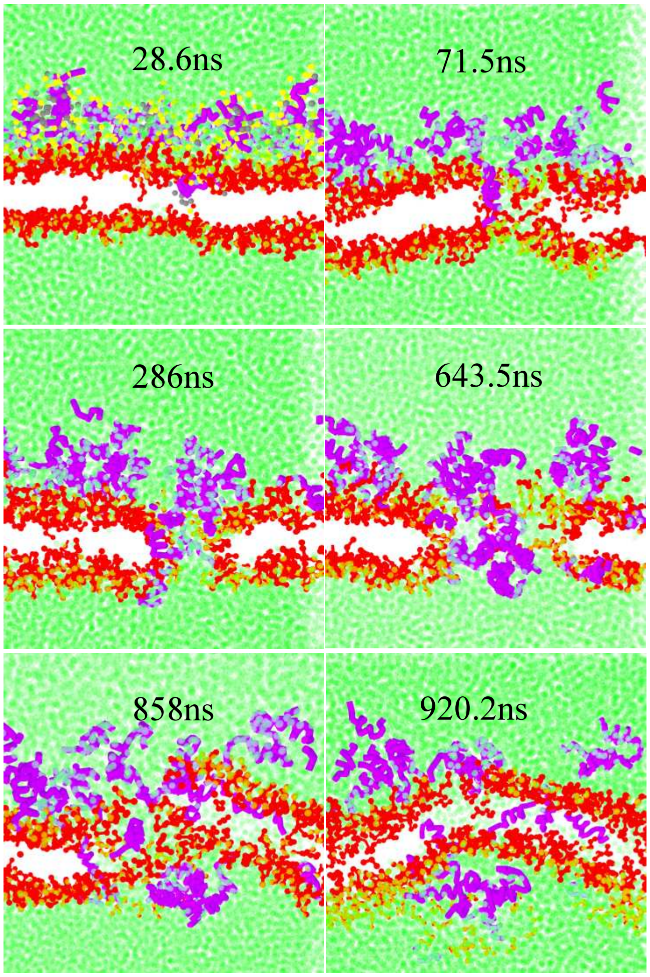

Figure S6. Evolution of a pore induced by 121 $hIAPP_{1-19}$ fragments in a bilayer membrane.

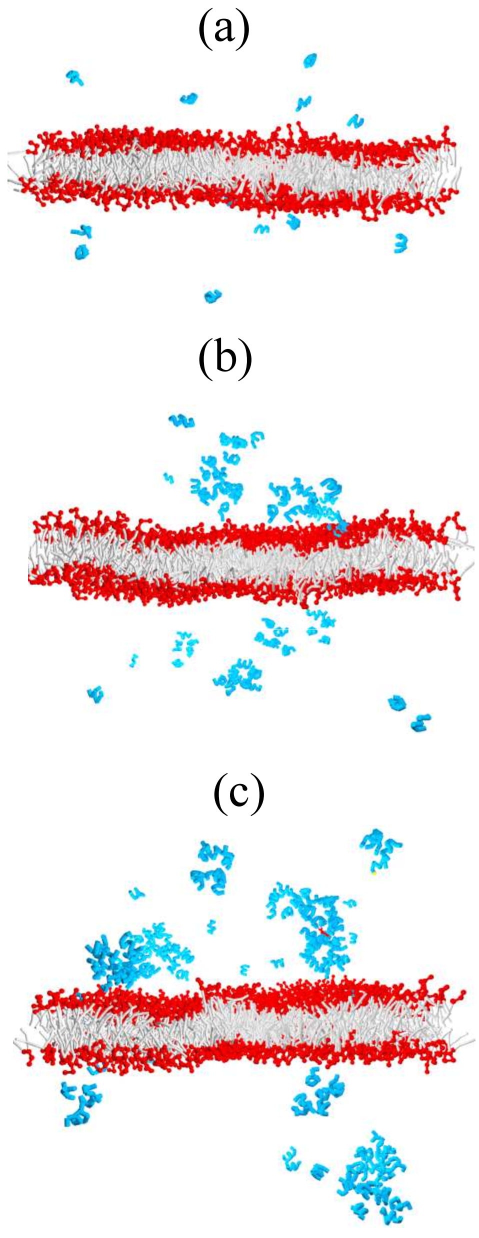

Figure S7. Snapshots of (a) 16, (b) 64, and (c) 121 hIAPP$_{20-29}$ fragments interacting with a bilayer membrane composed of 1600 lipids at a simulation time of 1.144 μs.

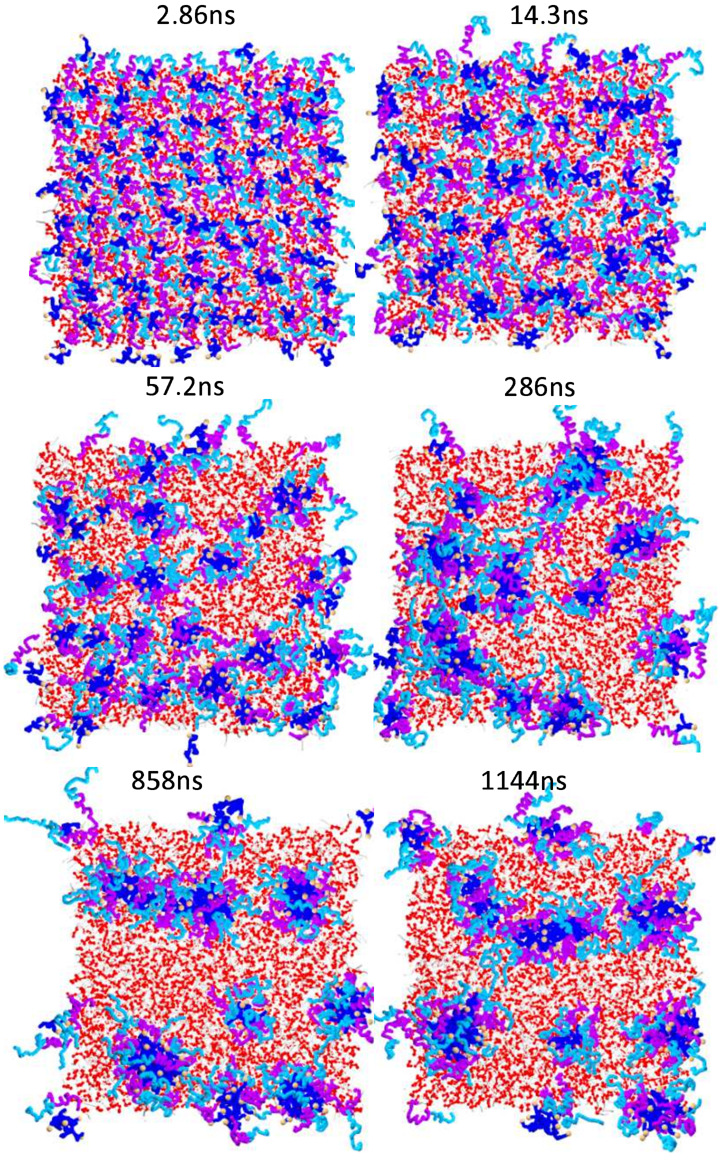

Figure S8. Time evalutional snapshots of 81 BSPOTPE and 81 hIAPP molecules interacting with a bilayer membrane composed of 1600 lipids.

References

1. Groot, R. D. & Warren, P. B. Dissipative particle dynamics: Bridging the gap between atomistic and mesoscopic simulation. *J. Chem. Phys.* **107**, 4423 (1997).
2. Groot, R. & Rabone, K. Mesoscopic simulation of cell membrane damage, morphology change and rupture by nonionic surfactants. *Biophys. J.* **81**, 725–736 (2001).
3. *http:://accelrys.com/products/materials-studio/* .
4. Nagle, J. F. & Tristram-Nagle, S. Structure of lipid bilayers. *Biochim. Biophys. Acta,Biomembr.* **1469**, 159–195 (2000).
5. Steltenkamp, S. *et al.* Mechanical properties of pore-spanning lipid bilayers probed by atomic force microscopy. *Biophys. J.* **91**, 217–226 (2006).
6. Marrink, S. J., de Vries, A. H. & Mark, A. E. Coarse grained model for semiquantitative lipid simulations. *J. Phys. Chem. B* **108**, 750–760 (2004).
7. Monticelli, L. *et al.* The martini coarse-grained force field: Extension to proteins. *J. Chem. Theory Comput.* **4**, 819–834 (2008).
8. de Jong, D. H., Lopez, C. A. & Marrink, S. J. Molecular view on protein sorting into liquid-ordered membrane domains mediated by gangliosides and lipid anchors. *Faraday Discuss.* **161**, 347–363 (2013).
9. Lopez, C. A., Sovova, Z., van Eerden, F. J., de Vries, A. H. & Marrink, S. J. Martini force field parameters for glycolipids. *J. Chem. Theory Comput.* **9**, 1694–1708 (2013).
10. Milano, G. & Muller-Plathe, F. Mapping atomistic simulations to mesoscopic models: a systematic coarse-graining procedure for vinyl polymer chains. *J. Phys. Chem. B* **109**, 18609–18619 (2008).
